# Supplementary material for: Assignment of individual structures from intermetalloid nickel gallium cluster ensembles
Source: Commun Chem. 2024 Feb 13;7:29. doi: 10.1038/s42004-024-01110-9 (PMC10864300; doi:10.1038/s42004-024-01110-9)
Supplement: Supplementary file 2 — Supporting Information [file 42004_2024_1110_MOESM2_ESM.pdf]

Supporting Information

Assignment of individual structures from intermetalloid nickel gallium cluster ensembles

Maximilian Muhr,[#,1] Johannes Stephan,[#,1] Lena Staiger,[1] Karina Hemmer,[1] Max Schütz,[1] Patricia Heiß,[1] Christian Jandl,[1] Mirza Cokoja,[1] Tim Kratky,[1] Sebastian Günther,[1] Dominik Huber,[1] Samia Kahlal,[2] Jean-Yves Saillard,*[,2] [c] Olivier Cador,[2] Augusto C. H. Da Silva,[3] Juarez L. F. Da Silva,*[,3] Janos Mink,[4] Christian Gemel,[1] Roland A. Fischer*[,1]

[1]Department of Chemistry and Catalysis Research Center, Technical University Munich, Lichtenbergstraße 4, D-85748 Garching, Germany

[2]Univ Rennes, CNRS, ISCR-UMR 6226, F-35000 Rennes, France.

[3]São Carlos Institute of Chemistry, University of São Paulo, P. O. Box 780, 13560-970, São Carlos, SP, Brazil.

[4]Hungarian Academy of Sciences, Institute of Material and Environmental Chemistry, Research Centre for Natural Sciences, Magyar tudósok körútja 2, H-1117 Budapest

The manuscript was written through contributions of all authors. / All authors have given approval to the final version of the manuscript.

[#] These authors contributed equally.

Abstract: Poorly selective mixed-metal cluster synthesis and separation yield reaction solutions of inseparable intermetalloid cluster mixtures, which are often discarded. High-resolution mass spectrometry, however, can provide precise compositional data of such product mixtures. Structure assignments can be achieved by advanced computational screening and consideration of the complete structural space. Here, we experimentally verify structure and composition of a whole cluster ensemble by combining a set of spectroscopic techniques. Our study case are the very similar nickel/gallium clusters of M_{12}, M_{13} and M_{14} core composition $Ni_{6+x}Ga_{6+y}$ ($x+y \leq 2$). The rationalization of structure, bonding and reactivity is built upon the organometallic superatom cluster $[Ni_6Ga_6](Cp^*)_6 = [Ga_6](NiCp^*)_6$ (**1**; $Cp^* = C_5Me_5$). The structural conclusions are validated by reactivity tests using carbon monoxide, which selectively binds to Ni sites, whereas (triisopropylsilyl)acetylene selectively binds to Ga sites.

Table of Contents

Supplementary Methods **1**
Synthesis procedures 1
Instrumentation 1
Crystallography 2
Density Functional Theory Calculations 2
Results and Discussion **3**
Analysis of cluster compounds 3
NMR MAS spectroscopy 3
NMR spectroscopy in solution 4
IR spectroscopy 5
Raman spectroscopy 6
LIFDI-MS measurements 9
Cluster Mixtures 19
Labelling and fragmentation experiments 20
SCXRD analysis 25
PXRD analysis 27
XPS measurements 28
Elemental Analysis 28
SQUID and EPR measurements 29
DFT calculations – Part 1 30
DFT calculations – Part 2 44
Bonding Details **44**
Computational Details 44
Reactivity Tests: CO **46**
NMR spectroscopy 46
IR spectroscopy 48
LIFDI-MS measurements 49
Supplementary References **51**

Supplementary Methods

Synthesis procedures

General Remarks. All experiments were carried out using standard Schlenk and glovebox techniques under an atmosphere of purified argon. Glassware was heated with hexamethyldisilazane to yield passivated surfaces. Toluene (Sigma-Aldrich, HPLC grade, 99.9%) and *n*-hexane (Sigma-Aldrich, HPLC grade, 99.9%) were dried using an MBraun Solvent Purification System (SPS) and stored over activated 3 Å molecular sieves. Mesitylene (Sigma-Aldrich, 99%) was dried by storing over activated 3 Å molecular sieves. The final water content of all solvents was checked by Karl-Fischer titration and did not exceed 5 ppm. $Ni(cod)_2$,[S1] $Ni_2(dvds)_3$,[S2] GaCp*[S3] and Ni(GaCp*)(dvds)[S2] were synthesised according to literature known procedures.

Synthesis of 1A – major component (3). $[Ni(cod)_2]$ (500 mg, 1.181 mmol) was suspended in toluene (5 ml) and $Ga(C_5Me_5)$ (435 mg, 2.122 mmol) was added at room temperature. The dark solution was heated to 65 °C for 2 days. Upon slow cooling to room temperature, a black solid (200 mg) was obtained, which was extracted with 16 ml of hot (100 °C) mesitylene. The hot mesitylene solution was concentrated under reduced pressure and analytically pure single crystals of **1A** were obtained upon slow cooling to room-temperature, isolated by means of cannula filtration and dried under reduced pressure to yield **1A** as black crystalline solid (155 mg).

Synthesis of 1B – major component (2).* $Ga(C_5Me_5)$ (482 mg, 1.07 mmol) was added to a solution of $[Ni(GaC_5Me_5)(dvds)]$ (290 mg, 1.42 mmol) in toluene (2 mL). After heating the dark red reaction solution to 110 °C for 3 h, the hot solution was filtered via a cannula. The solution was allowed to cool to ambient temperature overnight and a black precipitate was formed. This was separated from the reaction solution by means of cannula filtration. The residue was washed with small amounts of cold *n*-hexane and dried under reduced pressure, to yield **1B** as black crystalline solid (60.8 mg). **The synthesis can be performed also with* $[Ni_2(dvds)_3]$ *instead of* $[Ni(GaC_5Me_5)(dvds)]$*, while the Ni/Ga ratio is kept constant. Work up, yield, and spectroscopic results are identical.*

General Procedure for Reactions with CO. 10 mg of **1A** or **1B** were dissolved in 0.4 mL toluene-d_8 in a high-pressure J-Young NMR tube. The reaction mixture was pressurized with 1.0 bar CO at 25°C for 2 h. LIFDI-MS analysis, 0.1 ml of the reaction mixture was diluted with 0.2 mL toluene. For IR measurements, the reaction mixture dropped on the IR instrument and the solvent was evaporated.

Instrumentation

Elemental analyses and atom absorption spectrometry were performed by "Mikroanalytisches Laboratorium Kolbe", Oberhausen, Germany. NMR spectra of **1A** and **1B** were measured in C_6D_6 at 298 K, using a Bruker Advance DPX 250 or a Bruker Avance III AV400 US spectrometer operating at the appropriate frequencies. Chemical shifts are given relative to TMS, and spectra were referenced relative to the residual solvent signal. Solid-state magic angle spinning (MAS) NMR spectroscopic measurements of neat **1A** and **1B** were performed at room temperature in 4 mm ZrO_2 rotors (rotational frequency 15 kHz) on a Bruker Avance 300 spectrometer whereat spectra were referenced using the external standard adamantane (^1H: 2.00 ppm, ^{13}C: 29.47 ppm). FT-IR spectra were recorded on a Bruker Alpha FT-IR spectrometer with an ATR geometry, using a diamond ATR unit under argon atmosphere. The Raman spectra are recorded with an "InVia Raman Microscope" from Renishaw with a Newton EMCCD Camera (Spectroscopy EMCCD, 25.6 mm wide, 1600 pixel, 3 MHz) from the company Andor. A frequency-doubled Nd:YAG laser (532 nm) and an objective with 50x magnification (Leica N PLAN EPI 50x/0.75.) were used during the measurements. Unless otherwise noted, mass spectrometric data of diluted toluene solutions of **1A** and **1B** were acquired using a ThermoFisher Exactive Plus Orbitrap mass spectrometer equipped with a liquid injection field desorption ionization (LIFDI) source by Linden CMS GmbH. X-ray photoelectron spectra were recorded on a Leybold-Heraeus LHS 10 spectrometer using a non-monochromatized Al Kα source (1486.7 eV). Sample preparation and transfer into the XPS spectrometer were carried out under argon atmosphere. All spectra were recorded in an ultra-high vacuum chamber at a pressure below 5×10^{-8} mbar. The analyzer was operated at a constant pass energy of 100 eV leading to an energy resolution with a full width at half-maximum (fwhm) of ~1.1 eV. The energy scale of the spectra was corrected for sample charging by using the C 1s main signal (284.5 eV). Core level spectra were deconvoluted by using Voigt functions and linear background subtraction.

Crystallography

Powder X-ray diffraction measurements were performed on a Panalytical Empyrean instrument. The sample was put in a capillary under argon atmosphere and placed on a silicon wafer cut. The detection was carried out with a Pixel3D detector. All measurements were performed with Cu-Kα (λ = 1.54 Å) radiation and at 298 K.

Singe crystal X-Ray diffraction intensities for **1** were collected on a Bruker diffractometer equipped with a CMOS detector (APEX III, κ CMOS), a TXS rotating anode with Mo Kα radiation (λ = 0.71073 Å) and a Helios optic, or a Bruker diffractometer equipped with a CMOS detector (Bruker Photon 100), an IMS microsource with Cu Kα radiation (λ = 1.54178 Å) and a Helios mirror optic, respectively. Diffraction data was acquired and processed with the APEX-III program package.[S4] In a routine experiment, suitable crystals were coated in perfluoropolyether, and mounted in the cooled nitrogen stream of the diffractometer on a loop or microsampler. Lattice parameters were determined with an initial matrix scan. Reflections were merged and corrected for Lorenz and polarization effects, as well as scan speed and background using SAINT. Multiscan absorption corrections were performed with SADABS.[S5] The molecular structure was solved and refined with the program package SHELXLE, using the programs SHELXS-97 and SHELXL-2017.[S6-8] Hydrogen atoms were calculated in ideal positions as follows: Methyl hydrogen atoms were refined as part of rigid rotating groups, with a C–H distance of 0.98 Å and Uiso(H) = 1.5·Ueq(C). Other H atoms were placed in calculated positions and refined using a riding model, with methylene and aromatic C H distances of 0.99 Å and 0.95 Å, respectively, other C–H distances of 1.00 Å, all with Uiso(H) = 1.2·Ueq(C). Non-hydrogen atoms were refined with anisotropic displacement parameters. Full-matrix least-squares refinements were carried out by minimizing $\Sigma w(F_o^2 - F_c^2)^2$ with the SHELXL weighting scheme.[S8] Neutral atom scattering factors for all atoms and anomalous dispersion corrections for the non-hydrogen atoms were taken from International Tables for Crystallography.[S9] A split layer refinement was used for disordered groups and additional restraints on distances, angles and anisotropic displacement parameters were employed to ensure convergence within chemically reasonable limits. Images of the crystal structures were generated with Mercury.[S10]

Density Functional Theory Calculations

Part I (Screening): Our total energy calculations were based on spin-polarized density functional theory (DFT) within the semilocal exchange-correlation energy functional proposed by Perdew–Burke–Ernzerhof (PBE).[S11] The Kohn–Sham (KS) orbitals, which are required to solve the KS equations, were described by numeric atom-centered orbitals (NAO), as implemented in the all-electron Fritz–Haber Institute ab initio molecular simulations (FHI-aims) package.[S12] As provided within the FHI-aims package, we employed a minimal NAO basis set with a set of additional NAO added hierarchically up to the second basis set improvement, called light-tier2 in FHI-aims notation. For the relativistic corrections, we employed the scalar-relativistic framework with zero-order regular approximation (ZORA).[S13] Thus, the present framework, provides great flexibility to screen large number of complex trial configurations (geometric optimizations), which is required in the present study. For the self-consistency solution of the KS equations, we employed a total energy criterion of 10−5 eV, while the equilibrium geometries were obtained once the atomic forces were smaller than 10−2 eV Å−1 . For the vibrational frequency calculations, we decreased the forces criteria for 10−4 eV $Å^{-1}$ to calculate the Hessian matrix elements using atomic displacements of 2.5 × 10−3 Å. To avoid fractional occupation of the highest occupied molecular orbitals (HOMO) and lowest unoccupied molecular orbitals (LUMO), we employed a Gaussian broadening of 1 meV.

Part II (Bonding analysis): Density Functional Theory (DFT) calculations[S14] were carried out with the use of the Amsterdam Density Functional code (ADF2017)[S9] with the addition of Grimme's D3 empirical corrections[S15] in order to consider dispersion effects. The triple-ξ Slater basis set plus two polarization functions (STO-TZP),[S16] was used, together with the Becke-Perdew (BP86)[S16-17] exchange-correlation functional. All the optimized structures were confirmed as true minima on their potential energy surface by analytical vibration frequency calculations. The NMR chemical shifts were computed according to the gauge-independent atomic orbitals (GIAO) method,[S17] assuming the Zero Order Regular Approximation (ZORA) for clusters with S = 1/2.[S18]

Results and Discussion

Analysis of cluster compounds

NMR MAS spectroscopy

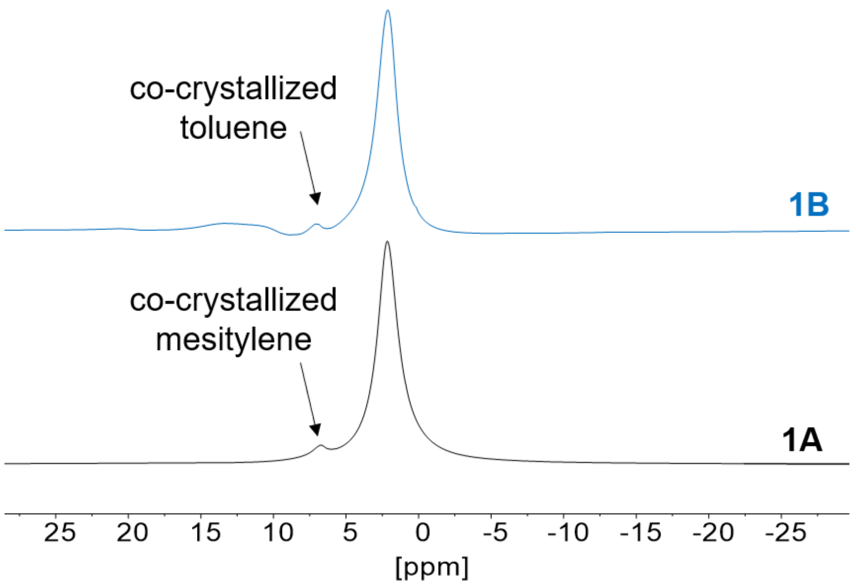

Figure S1. ^{1}H MAS NMR spectra of **1A** (**3** major component) and **1B** (**2** major component) showing a broad signal at 2.15 ppm assigned to the Cp* shell. δ [ppm] = 6.71 (mesitylene, CH), 2.04 (s, Cp*).

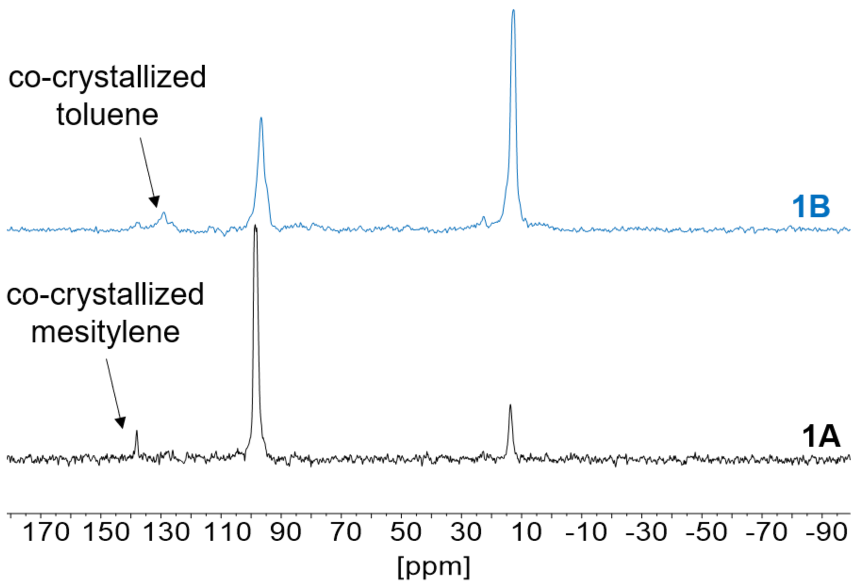

Figure S2. ^{13}C MAS NMR spectra of **1A** (**3** major component) and **1B** (**2** major component). Top, blue ^{13}C NMR: δ = 129.0 (toluene, ArC), 96.9 (Cp*, ring), 12.3 (Cp*, CH_3) ppm. Bottom, black ^{13}C NMR: δ = 138.1 (mesitylene, ArC), 98.7 (Cp*, ring), 13.7 (Cp*, CH_3) ppm. The ^{13}C Cp* ring shifts fit well to shifts of the NiCp* signals of [(μ_2-Ga(C_5Me_5))(Ni_2)(μ_2-GaNi(C_5Me_5))$_2$(dvds)$_2$] (Figure S3). Despite the paramagnetism of **3** (**1A** major component) shifts of **2** and **3** are very similar and in a common range. This phenomena has been observed before for bigger metal clusters, like for the polyradical cluster [$Cu_{43}Al_{12}$](Cp*)$_{12}$.

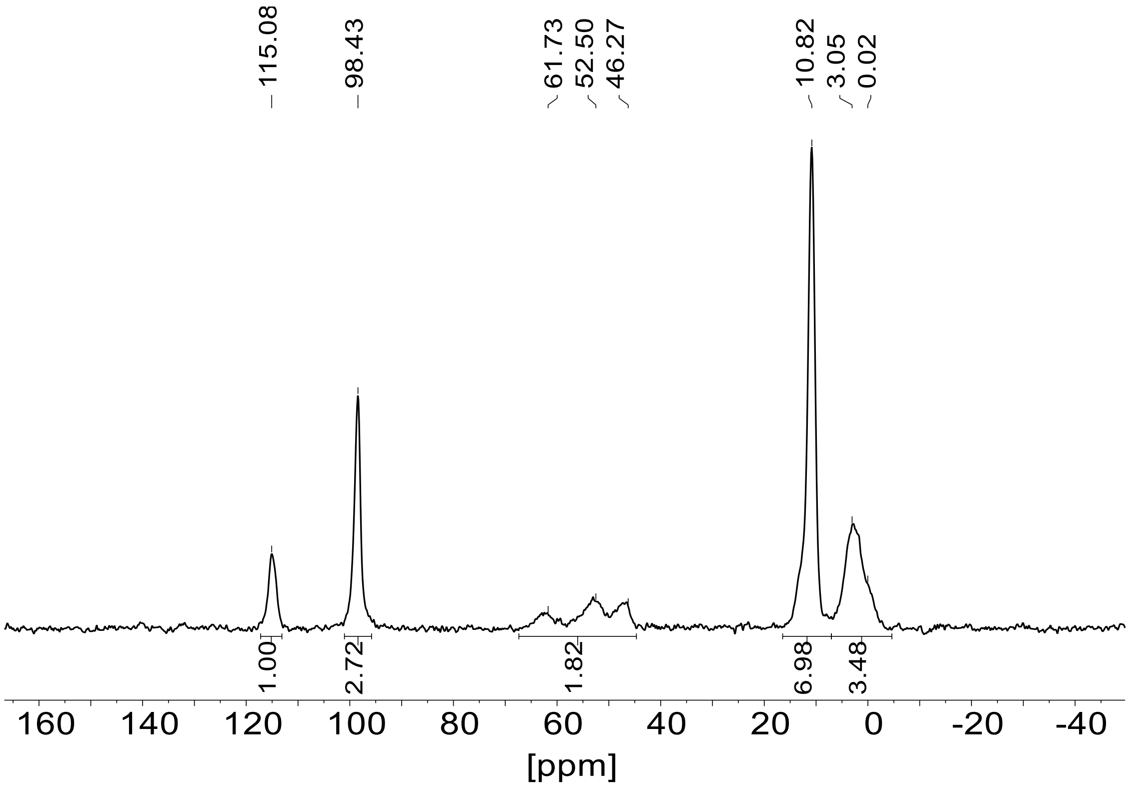

Figure S3. ^{13}C MAS NMR spectra of [(μ$_2$-Ga(C_5Me_5))(Ni_2)(μ$_2$-GaNi(C_5Me_5))$_2$(dvds)$_2$]. ^{13}C NMR: δ = 115.08 (**Ga**Cp*, ring), 98.43 (**Ni**Cp*, ring), 61.73 – 46.27 (dvds, HC=CH_2), 10.82 (Cp*, CH_3), 3.05 – 0.02 (dvds, CH_3) ppm.

NMR spectroscopy in solution

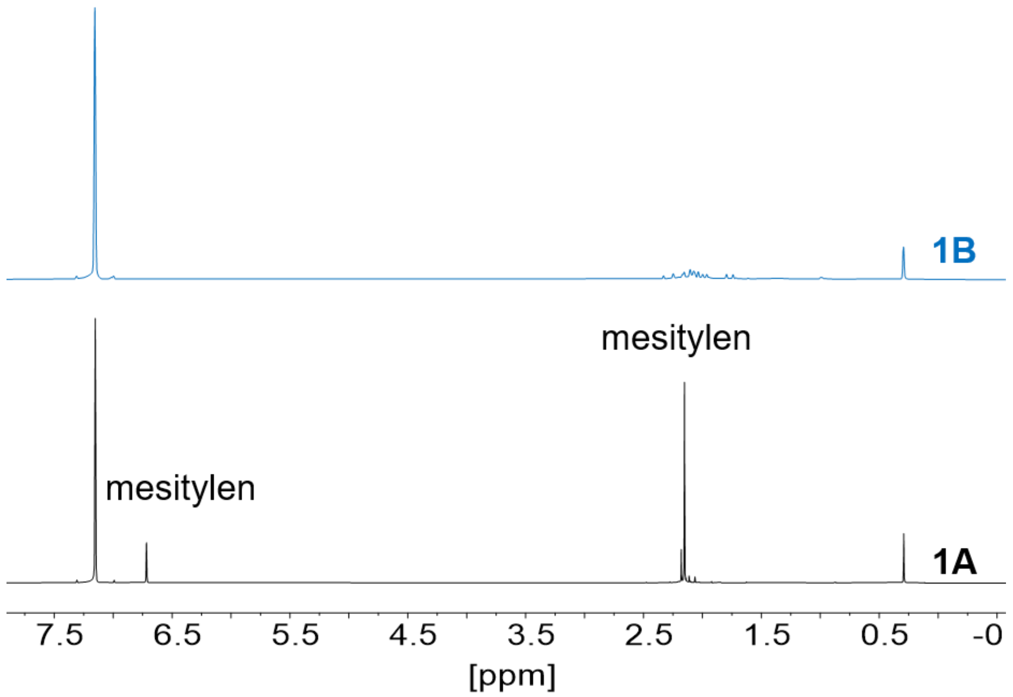

Figure S4. ^{1}H NMR in toluene-d_8 for **1A** (**3** major component) and **1B** (**2** major component). For **1B**, residual signals of dvds are apparent. For both mixtures, no cluster signals are detected due to its bad solubility.

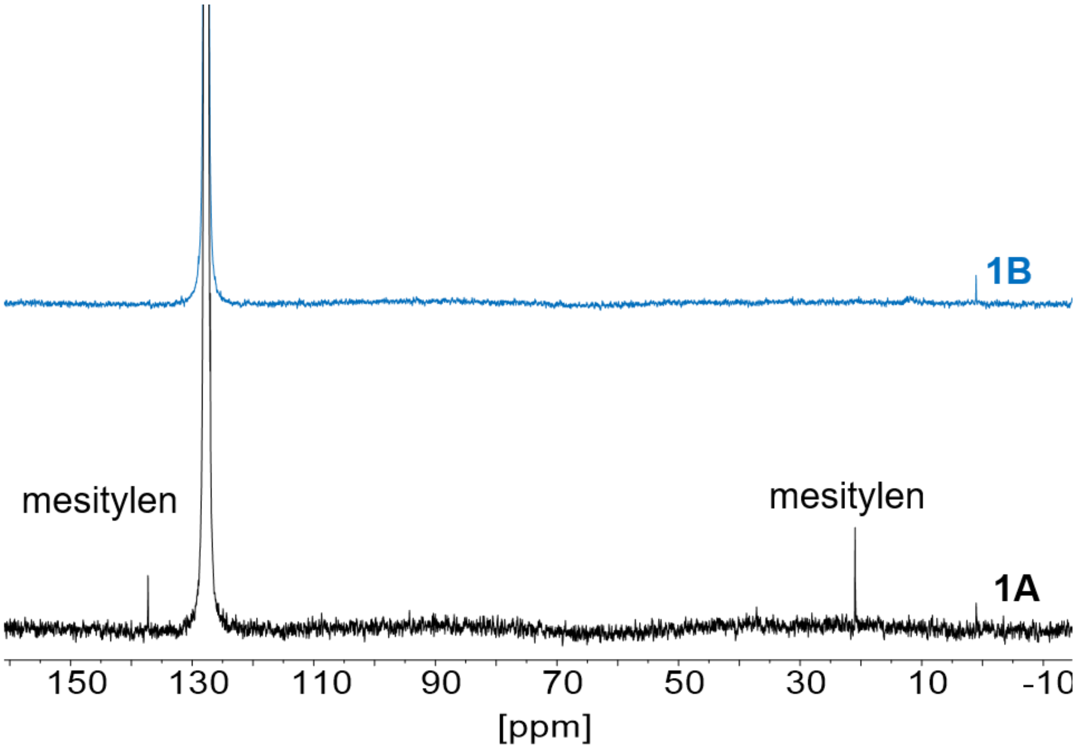

Figure S5. ^{13}C NMR in toluene-d_8 for **1A** (**3** major component) and **1B** (**2** major component). For both mixtures, no cluster signals are detected due to its bad solubility.

IR spectroscopy

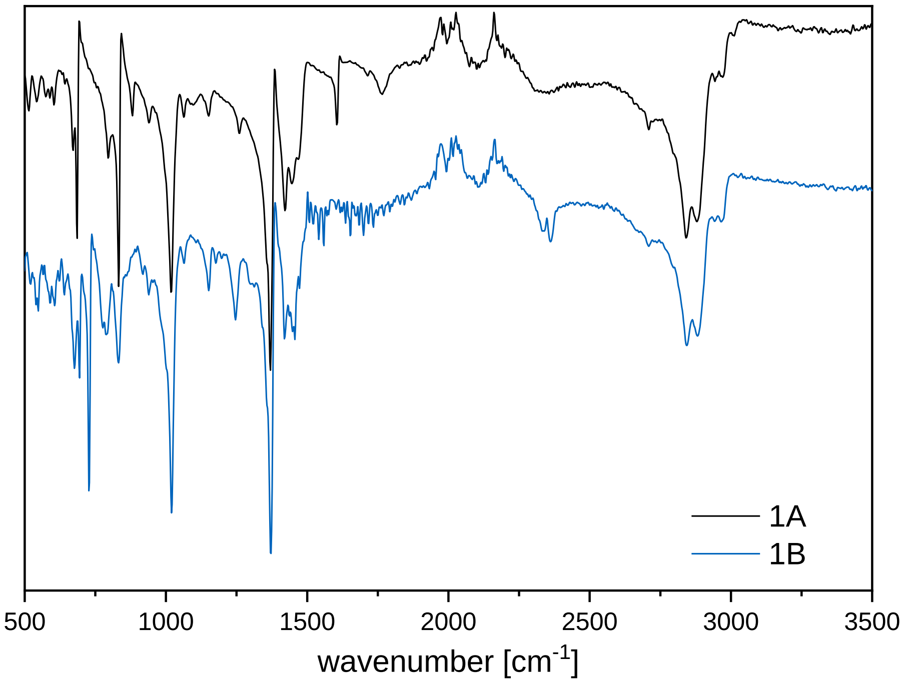

Figure S6. FT-ATR-IR spectra of **1A** (**3** major component) and **1B** (**2** major component). IR (ATR, neat, cm^{-1}): 2968, 2881, 2843, 2705, 1460, 1422, 1372, 1022, 728, 1153, 1072, 947, 797, 696, 671, 596, 546, 465.

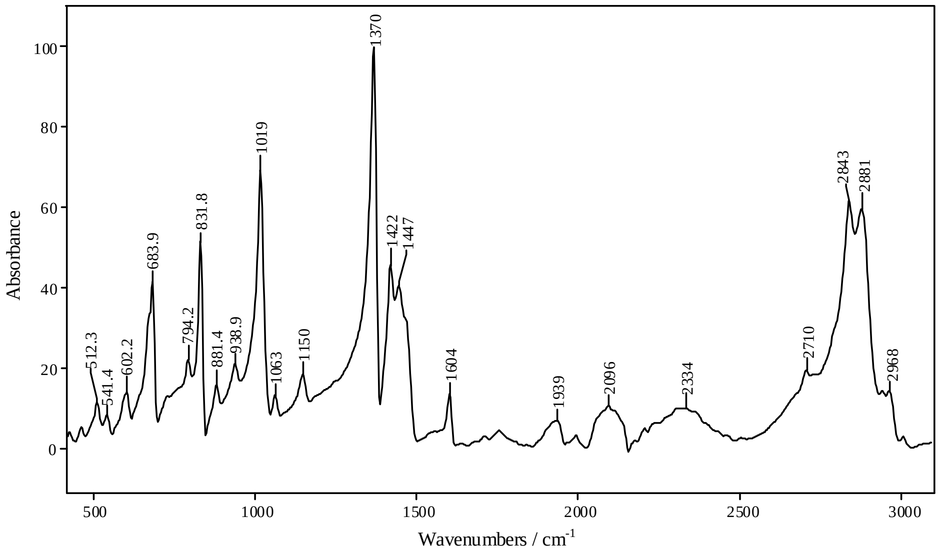

Figure S7. Mid-infrared ATR spectrum of solid **1A** (**3** major component) sample without ATR correction. The band intensities are normalized to the strongest band at 1370 cm^{-1}, taken as 100 arbitrary intensity units. (The experimentally recorded intensity of this band was 0.14 absorbance units).

Raman spectroscopy

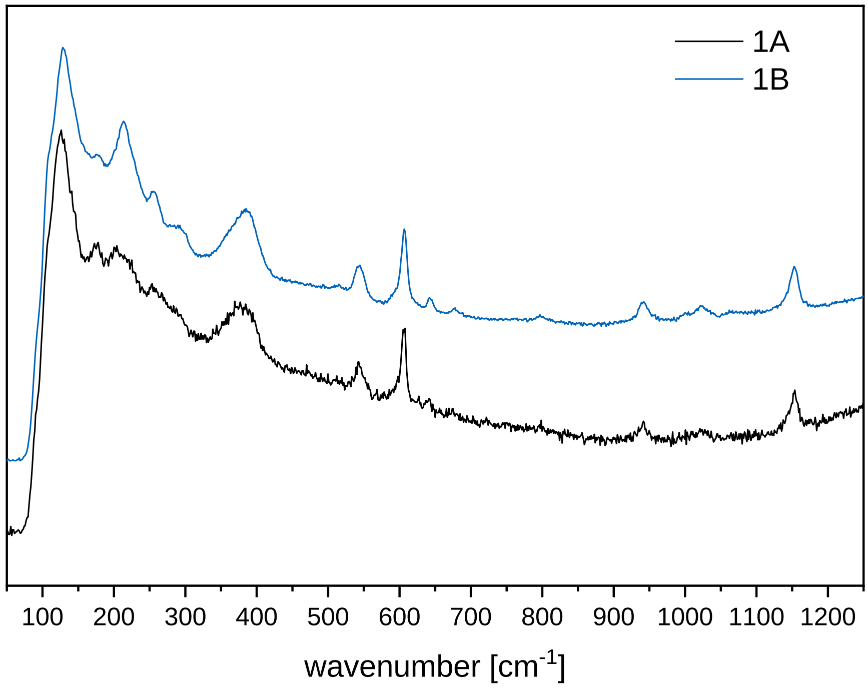

Figure S8. Raman spectra of **1A** (**3** major component) and **1B** (**2** major component).

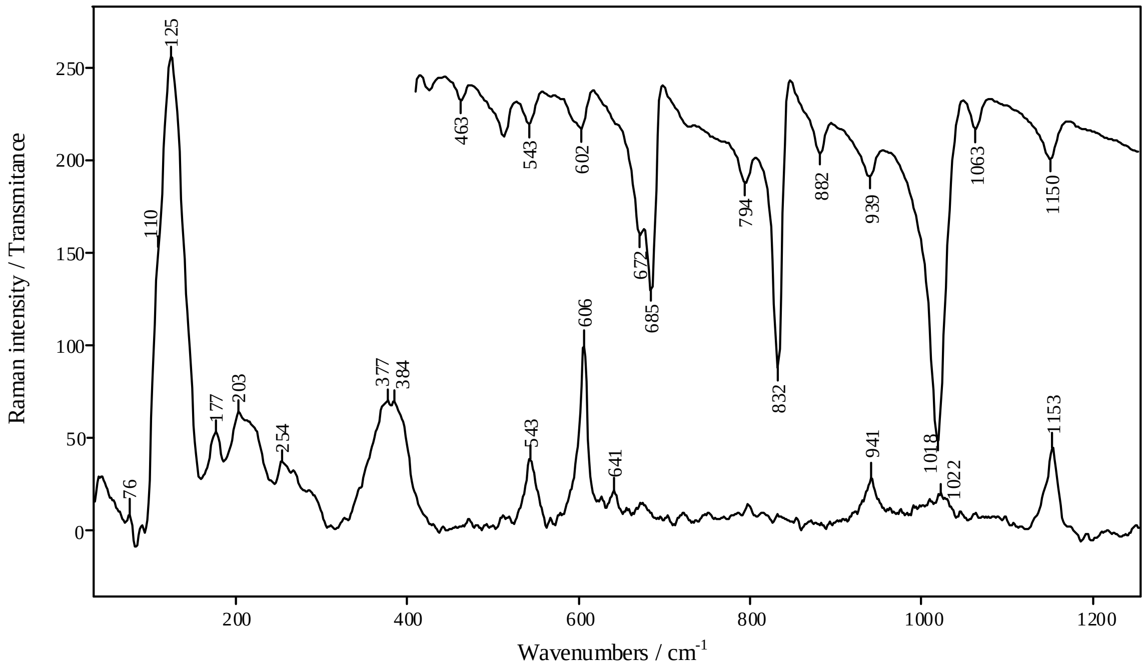

Figure S9. Mid-infrared ATR spectrum (upper trace) of solid **1A** (**3** major component) without ATR correction and *Raman* spectrum (lower trace). The IR band intensities are normalized to the strongest band at 1370 cm^{-1}, taken as 100 arbitrary intensity units. *Raman* band intensities are normalized to the strong band at 606 cm^{-1}, taken as 100 intensity units.

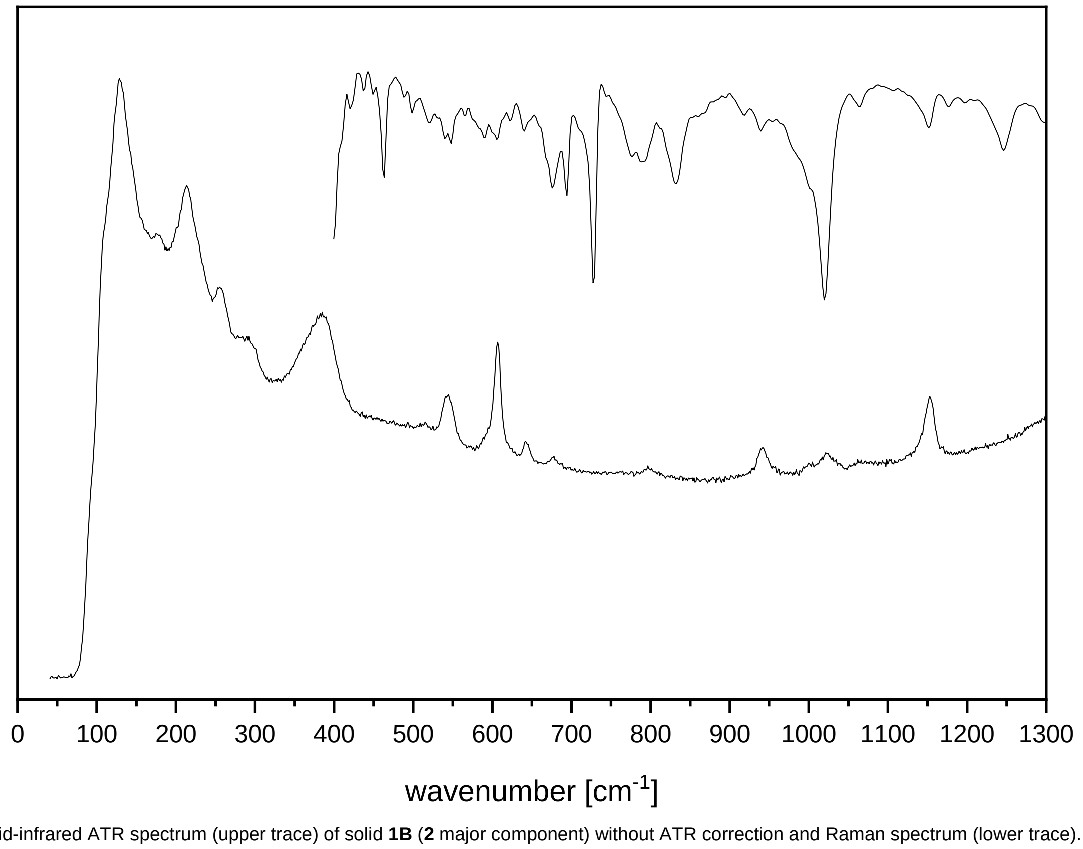

Figure S10. Mid-infrared ATR spectrum (upper trace) of solid **1B** (**2** major component) without ATR correction and Raman spectrum (lower trace).

Table S1: Experimental infrared and Raman frequencies (cm^{-1}) and their tentative assignments for **1A**.

**Infrared**	**Raman**	**Suggested assignments and their notations**
3011(3)[a]		
2968(15)<br>2945(15)		CH_3 asym stretch (n_aCH_3)
2881(60)<br>2843(63)		CH_3 sym stretch (n_sCH_3)
2797(33)sh<br>2736(24)sh<br>2709(26)<br>2365(4)<br>1759(6)		Combination bands and overtones<br>2 x 1370 (2 x d_sCH_3)
1605(15)		
1468(30)<br>1447(39)		CH_3 asym deform (d_aCH_3)
1422(49)		$n_1(A_1)$ [b] ring sym CC stretch and C-Me stretch (n_sCC)<br>$n_6(E_1)$ n_aCC ring
1370(100)[c]		CH_3 sym deform umbrella (d_aCH_3); $n_{11}(E_2)$ ring CC asym stretch + C-Me asym stretch
1247vw[d]<br>1150(16)	 1153(44)<br>1134w,sh	 $n_{12}(E_2)$ (?)
1063(10)	1061(4)<br>1046(5)	$n_{12}(E_2)$ ring CC stretch + C-Me stretch + CH_3 rocking (rCH_3)
1019(63)<br>990 w,sh<br>939(19)	1022(20)<br>991(5)<br>941(38)	CH_3 rocking (rCH_3)
882(15)<br>860w,sh	871(5), b<br>854(7)	$n_7(E_1)$ (?)
832(53)	832(5)<br>815(5)	$n_7(E_1)$ ring CC asym stretch (n_aCC)
794(30)<br>758w,b<br>726w,sh	798(14)<br>750(4)<br>723(9)<br>704(5)	
685(40)<br>672(30)	 674(15)	$n_{16}(E_2)$ ring in plane deform (dCCC)
626w,sh<br>603(11)	627(15)<br>606(100)[c]	$n_2(A_1)$ ring CC sym stretch (n_sCC)
593w,sh<br>567vw	590(30)sh<br>567(8)	
543(10)<br>513(12)	543(38)<br>515(7)	$n_5(A_2)$ C-Me in plane deform (bCMe)
497w,sh	492(3)	
 463(4)	472(6)<br>462w,sh<br>442(3)<br>430(4)	$n_{14}(E_2)$ ring CCC in plane deform (dCCC)
	384(70)	$n_4(A_1)$ Ni-Cp* sym stretch (n_s Ni-Cp*)
	286(31)<br>267(30)<br>253(37)	$n_{10}(E_1)$ Ni-Cp* asym stretch (tilt mode) (n_aNi-Cp*)
	217(53)sh<br>203(64)	$n_3(A_1)$ C-Me out of plane deform (gCMe)<br>$n_8(E_1)$ C-Me in plane deform (bCMe)
	177(53)	$n_{13}(E_2)$ C-Me in plane deform (bCMe)
	126(250)	$n_9(E_1)$ C-Me out of plane deform (gCMe)
	110(150)sh	$n_{15}(E_2)$ C-Me oop deform (gCMe)
	177(53)<br>177 (250)<br>110(150)sh	Ni-Ga, Ga-Ga stretching modes
	90(3)<br>76(7)<br>43(3)	Cluster skeletal deformations and lattice modes

Remarks*:*

[a] Relative band intensities are in brackets; [b] Selected bands for 100 intensity units; [c] Abbreviation of week bands and band shapes; w, weak; vw, very weak; b, broad; sh, shoulder.

LIFDI-MS measurements

Isolated Clusters

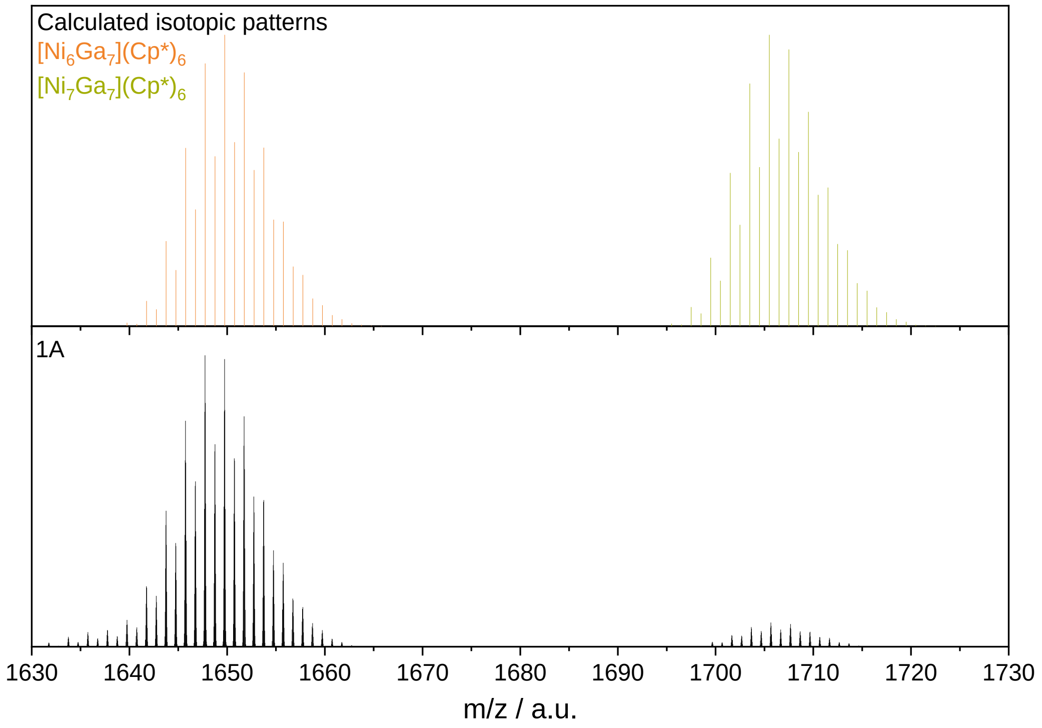

Figure S11. Relevant cut out of LIFDI mass spectra of cluster mixtures **1A** (**3** major component) exhibiting molecular ion signals of $[Ni_6Ga_7](Cp^*)_6$ (orange) and $[Ni_7Ga_7](Cp^*)_6$ (green).

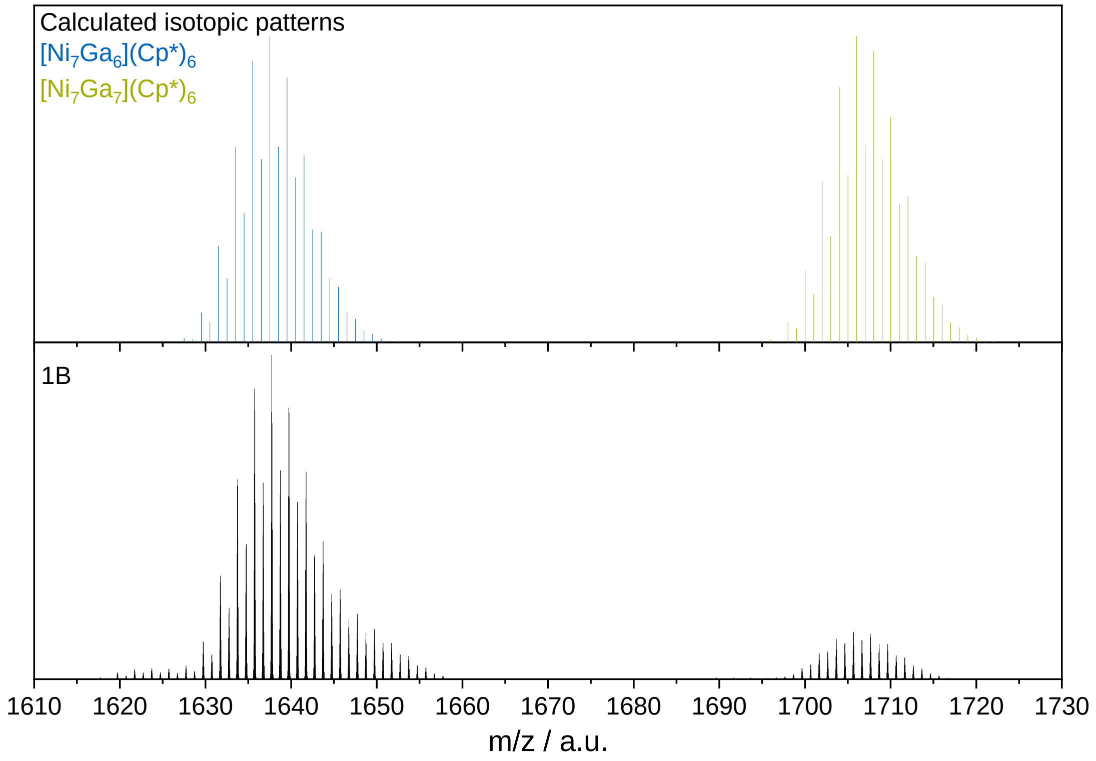

Figure S12. Relevant cut out of LIFDI mass spectra of cluster mixtures **1B** (**2** major component) exhibiting molecular ion signals of $[Ni_7Ga_6](Cp^*)_6$ (blue) and $[Ni_7Ga_7](Cp^*)_6$ (green).

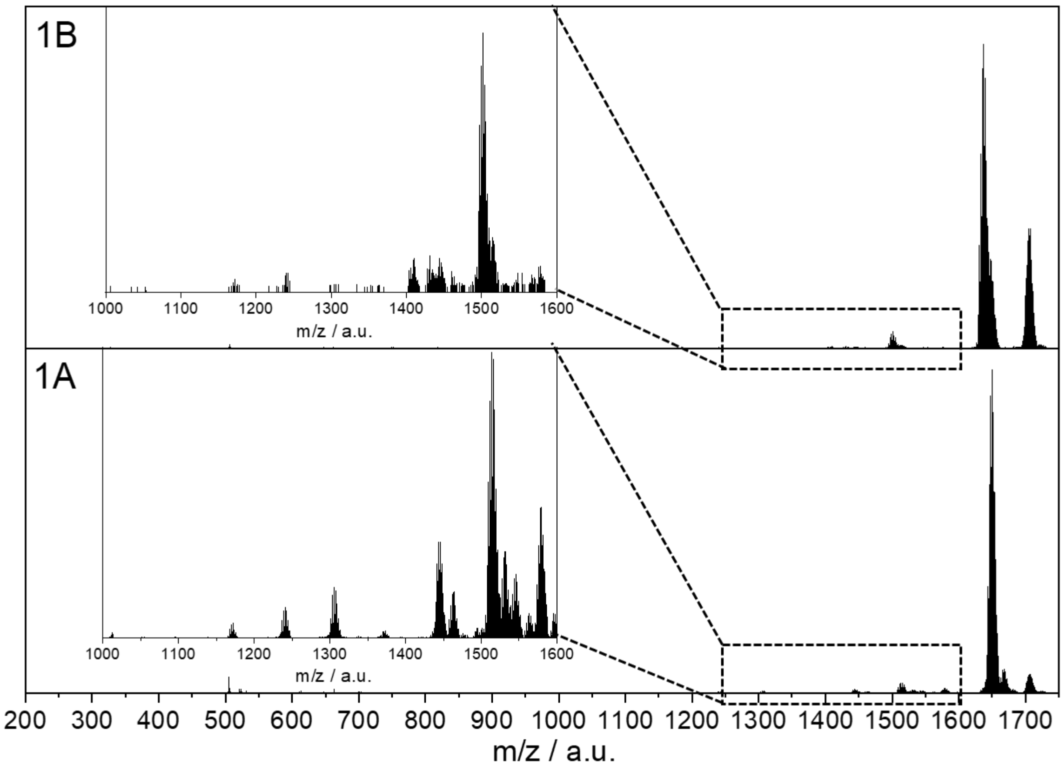

Figure S13. Full LIFDI mass spectra of cluster mixtures **1A** (major component **3**, bottom) and **1B** (major component **2**, top). *m/z* = 1639.5 ([$Ni_7Ga_6Cp^*_6$]$^+$, calc. 1638.6), 1649.3 ([$Ni_6Ga_7Cp^*_6$]$^+$, calc. 1650.3), 1706.7 ([$Ni_7Ga_7Cp^*_6$]$^+$, calc. 1706.7) with zoom-in for a better depiction of signals < 1400 *m/z*.

Table S2: Measured clusters and related mass shifts by H-atom and C_5Me_5 fragmentation including reactivity tests.

**Measured m/z**	**Measured cluster**	**Attributed cluster**
328.1694	[$Ni(C_5Me_5)_2$]$^+$	[$Ni(C_5Me_5)_2$]
750.2112	[$(Ni_7Ga_6)(C_5Me_5)_5$]$^{2+}$ **-3H**	[$(Ni_7Ga_6)(C_5Me_5)_6$]
819.3131	[$(Ni_7Ga_6)(C_5Me_5)_6$]$^{2+}$ **-2H**	[$(Ni_7Ga_6)(C_5Me_5)_6$]
877.3982	[$Ni_4Ga_3(C_5Me_5)_3(CO)$]$^+$	[$Ni_4Ga_3(C_5Me_5)_3(CO)$]
933.4577	[$Ni_3Ga_4(C_5Me_5)_3(CO)_3$]$^+$	[$Ni_3Ga_4(C_5Me_5)_3(CO)_3$]
989.2956	[$Ni_4Ga_3(C_5Me_5)_3(CO)_5$]$^+$	[$Ni_4Ga_3(C_5Me_5)_3(CO)_5$]
1166.6057	[$Ni_4Ga_4(C_5Me_5)_4(CO)_4$]$^+$	[$Ni_4Ga_4(C_5Me_5)_4(CO)_4$]
1194.5725	[$Ni_4Ga_4(C_5Me_5)_4(CO)_5$]$^+$	[$Ni_4Ga_4(C_5Me_5)_4(CO)_5$]
1237.3485	[$Ni_6Ga_5(C_5Me_5)_4$]$^+$ **-3H**	[$Ni_6Ga_5(C_5Me_5)_6$]
1306.3661	[$Ni_6Ga_6(C_5Me_5)_4$]$^+$ **-4H**	[$Ni_6Ga_6(C_5Me_5)_6$]
1444.0381	[$Ni_6Ga_6(C_5Me_5)_5$ $^+$ **-H**	[$Ni_6Ga_6(C_5Me_5)_6$]
1503.0914	[$Ni_7Ga_6(C_5Me_5)_5$]$^+$ **-3H**	[$Ni_7Ga_6(C_5Me_5)_6$]
1513.9057	[$Ni_6Ga_7(C_5Me_5)_5$]$^+$ **-2H**	[$Ni_6Ga_7(C_5Me_5)_6$]
1568.9456	[$(NiGa_5)(NiC_5Me_5)_6$]$^+$ **-3H**	[$(NiGa_5)(NiC_5Me_5)_6$]
1579.3361	[$(Ga_6)(NiC_5Me_5)_6$]$^+$ **-2H**	[$(Ga_6)(NiC_5Me_5)_6$]
1639.5566	[$(NiGa_6)(NiC_5Me_5)_6$]$^+$ **-2H**	[$(NiGa_6)(NiC_5Me_5)_6$]
1649.3240	[$(Ga_7)(NiC_5Me_5)_6$]$^+$ **-H**	[$(Ga_7)(NiC_5Me_5)_6$]
1706.7280	[$(NiGa_7)(NiC_5Me_5)_6$]$^+$ **-3H**	[$(NiGa_7)(NiC_5Me_5)_6$]
1707-1720	[$(NiGa_7)(NiC_5Me_5)_6H_x$]$^+$	[$(NiGa_7)(NiC_5Me_5)_6H_x$]
1736.3855	[$(OC)(NiGa_7)(NiC_5Me_5)_6$]$^+$ **-H**	[$(OC)(NiGa_7)(NiC_5Me_5)_6$]

Table S3: Tabular pattern of [$Ni(C_5Me_5)_2$].

m/z	relative intensity
327,15701	37.93278
328,16404	100
329,16723	19.61349
330,15796	36. 38609
331,1646	7.58823

Table S4: Tabular pattern of [$(Ni_7Ga_6)(C_5Me_5)_5]^{2+}$.

m/z	relative intensity
745,80136	17.28214
746,30392	7.71162
746,80698	37.57649
747,31055	20.74337
747,81464	58.15704
748,31923	29.88889
748,81461	79.66233
749,32022	49.39291
749,82633	95.72439
750,32322	55.73508
750,82059	100
751,31846	54.4833
751,8266	78.69696
752,32548	41.59237
752,82484	47.76618
753,32471	20.88495
753,82507	25.83276
754,32594	7.04958
754,81746	6.00376

Table S5: Tabular pattern of [$(Ni_7Ga_6)(C_5Me_5)_6]^{2+}$.

m/z	relative intensity
816,89204	50.69447
817,39056	23.0507
817,88954	77.38423
818,38898	37.35102
818,88887	100
819,38922	52.53035
819,89003	81.15509
820,3913	48.33168
820,89303	54.86947
821,38405	24.85441
821,88669	20.98484
822,3898	6.66308
822,88216	10.80773

Table S6: Tabular pattern of $[Ni_4Ga_3(C_5Me_5)_3(CO)]^+$.

m/z	relative intensity
873,85517	58.76044
874,86066	16.6387
875,85558	100
876,86452	32.12259
877,85052	92.31414
878,86291	30.05687
879,85229	63.71766
880,85575	17.21035
881,8485	26.51345
873,85517	58.76044
874,86066	16.6387
875,85558	100
876,86452	32.12259
877,85052	92.31414
878,86291	30.05687
879,85229	63.71766
880,85575	17.21035
881,8485	26.51345

Table S7: Tabular pattern of $[Ni_4Ga_3(C_5Me_5)_3(CO)]^+$.

m/z	relative intensity
927,85029	16.66429
928,85642	5.04295
929,85075	64.47679
930,86014	22.27274
931,8442	100
932,85684	37.42308
933,84407	97.40764
934,84642	36.92606
935,85038	58.68291
936,84236	23.04152
937,83592	28.04804
938,84471	10.69985
939,84145	10.1901
940,83979	5.1593

Table S8: Tabular pattern of $[Ni_4Ga_3(C_5Me_5)_3(CO)_5]^+$.

m/z	relative intensity
985,84822	60.61613
986,84725	17.13547

987,83308	100
988,83514	34.33648
989,83871	97.8972
990,84382	35.30131
991,83565	59.49191
992,82896	15.3932
993,83863	21.93666

Table S9: Tabular pattern of $[Ni_4Ga_4(C_5Me_5)_4(CO)_4]^+$.

m/z	relative intensity
1161,88361	45.36654
1162,89857	21.68904
1163,89602	86.79557
1164,89476	43.87516
1165,89479	100
1166,89611	49.3013
1167,87978	83.13031
1168,88366	39.98315
1169,88883	46.22334
1170,87629	21.47925
1171,88403	20.9447
1172,87402	9.101
1173,88435	7.35934

Table S10: Tabular pattern of $[Ni_4Ga_4(C_5Me_5)_4(CO)_5]^+$.

m/z	relative intensity
1189,87819	41.57827
1190,89107	19.46198
1191,88574	85.48513
1192,88165	41.27225
1193,87881	100
1194,87722	47.8827
1195,87688	79.99068
1196,8778	38.12319
1197,87998	46.5697
1198,88341	19.94846
1199,86839	18.99554
1200,87433	7.61123
1201,88153	5.51062

Table S11: Tabular pattern of $[(Ni_6Ga_5)(C_5Me_5)_4]^+$.

m/z	relative intensity
1231,64151	42.66806
1232,64663	13.85806
1233,65298	69.96209
1234,63998	34.84223
1235,64877	92.64551
1236,6588	50.97934
1237,64942	100
1238,6619	46.77776
1239,65492	82.11756
1240,64914	35.3182
1241,66531	46.75654
1242,66194	22.06385
1243,65978	21.8133
1577,56791	10.56862
1578,54904	5.18729

Table S12: Tabular pattern of $[(Ni_6Ga_5)(C_5Me_5)_4]$.

m/z	relative intensity
1299,55839	30.94977
1300,55881	15.95074
1301,56039	60.02883
1302,56313	32.42021
1303,56703	93.05909
1304,57209	50.80303
1305,57832	100
1306,5633	54.87798
1307,57183	91.3854
1308,55908	47.4851
1309,56992	70.09436
1310,55943	30.98551
1311,57259	38.86939
1312,56437	18.86574
1313,55727	17.742
1314,57391	6.85318
1315,5691	6.81482

Table S13: Tabular pattern of $[(Ni_6Ga_6)(C_5Me_5)_5]^+$.

m/z	relative intensity
1437,70467	18.6758

1438,71352	29.19795
1439,69753	55.74515
1440,70847	60.86807
1441,69452	85.80158
1442,70758	80.38935
1443,69568	100
1444,71084	80.60071
1445,70100	82.86013
1446,71828	63.09803
1447,71051	68.1918
1448,70376	36.56838
1449,69803	28.72266
1450,69332	8.96359
1451,68965	7.35914

Table S14: Tabular pattern of $[(Ni_7Ga_6)(C_5Me_5)_5]^+$.

m/z	relative intensity
1497,60857	29.69234
1498,59859	8.12387
1499,61714	36.13902
1500,63672	8.7817
1501,62974	41.27537
1502,62375	8.8802
1503,61875	39.21342
1504,64242	22.27703
1505,63942	100
1506,63741	28.44248

Table S15: Tabular pattern of $[(Ni_6Ga_7)(C_5Me_5)_5]^+$.

m/z	relative intensity
1507,6364	33.57786
1508,63638	22.04713
1509,63736	67.2132
1510,63933	40.5724
1511,6423	88.86922
1512,61837	53.44879
1513,62331	100
1514,62926	60.84805
1515,63621	83.02229
1516,61615	45.44515
1517,62508	59.89516
1518,63501	28.86777

1519,61786	31.09466
1520,62979	15.53685
1521,61458	11.94242

Table S16: Tabular pattern of [$(NiGa_5)(NiC_5Me_5)_6$].

m/z	relative intensity
1560,55985	9.20692
1561,55442	20.34585
1562,54993	36.94623
1563,5464	43.7215
1564,54382	66.67271
1565,5422	69.97564
1566,54153	86.48722
1567,54182	88.79004
1568,54307	100
1569,54528	91.27486
1570,54844	91.6635
1571,55257	78.68838
1572,55767	67.81736
1573,56373	49.88777
1574,54112	41.67222
1575,54908	27.38829
1576,55801	18.49319
1577,56791	10.56862
1578,54904	5.18729

Table S17: Tabular pattern of [$(Ga_6)(NiC_5Me_5)_6$].

m/z	relative intensity
1571,84759	31.24904
1572,85297	14.12468
1573,8297	43.56054
1574,86662	17.28074
1575,84523	80.36374
1576,85444	39.45797
1577,83491	100
1578,84603	56.47025
1579,85814	97.14968
1580,8414	56.72672
1581,82558	68.13657
1582,84055	37.02157
1583,8266	37.88993
1584,8435	9.67171
1585,83142	8.97115

Table S18: Tabular pattern of **1B**.

m/z	relative intensity
1629,73434	11.58002
1630,76454	7.67025
1631,76446	31.93859
1632,76529	21.99933
1633,76705	61.64393
1634,76972	41.65335
1635,74195	89.66781
1636,74644	60.61426
1637,75187	100
1638,75822	64.44706
1639,734	83.70823
1640,74218	54.66276
1641,75128	64.00252
1642,76132	38.40984
1643,74068	42.48728

Table S19: Tabular pattern of **1A**.

m/z	relative intensity
1639,74946	9.22273
1640,75765	6.6503
1641,73523	20.7523
1642,74526	17.4663
1643,75622	46.64255
1644,73648	35.59128
1645,74928	77.53418
1646,76301	56.74664
1647,74596	100
1648,76154	69.47934
1649,74628	98.72563
1650,7319	64.63276
1651,75025	79.04945
1652,73767	51.51337
1653,75787	50.32614
1654,74709	33.08134
1655,73719	28.79991
1656,72819	16.50902
1657,75209	13.61196
1658,74489	8.10834
1659,73859	5.67744

Table S20: Tabular pattern of $[(NiGa_7)(NiC_5Me_5)_6]$.

m/z	relative intensity
1697,65659	5.28835
1698,65226	10.12338
1699,68203	23.98616
1700,67948	30.90347
1701,6778	55.1649
1702,67701	58.46905
1703,6771	85.18713
1704,67806	76.55479
1705,67991	100
1706,64921	82.74693
1707,65279	95.6326
1708,65727	74.27194
1709,66263	74.1018
1710,66887	50.20393
1711,67601	45.4295
1712,65042	29,08788
1713,65931	23.24051
1714,66909	12.0013
1715,64605	7.66613

Table S21: Tabular pattern of $[(OC)(NiGa_7)(NiC_5Me_5)_6]$.

m/z	relative intensity
1729,71408	32.62559
1730,70393	18.98724
1731,69464	64.33649
1732,7204	37.52542
1733,71284	90.225
1734,70614	57.06226
1735,70028	100
1736,69528	58.90946
1737,69114	86.8921
1738,68785	49,3679
1739,68542	61.50577
1740,68385	31.82106
1741,68314	37.1474
1742,68329	16.39531
1743,68431	18.84153
1744,68618	7.55527
1745,65433	9.1702

Cluster Mixtures

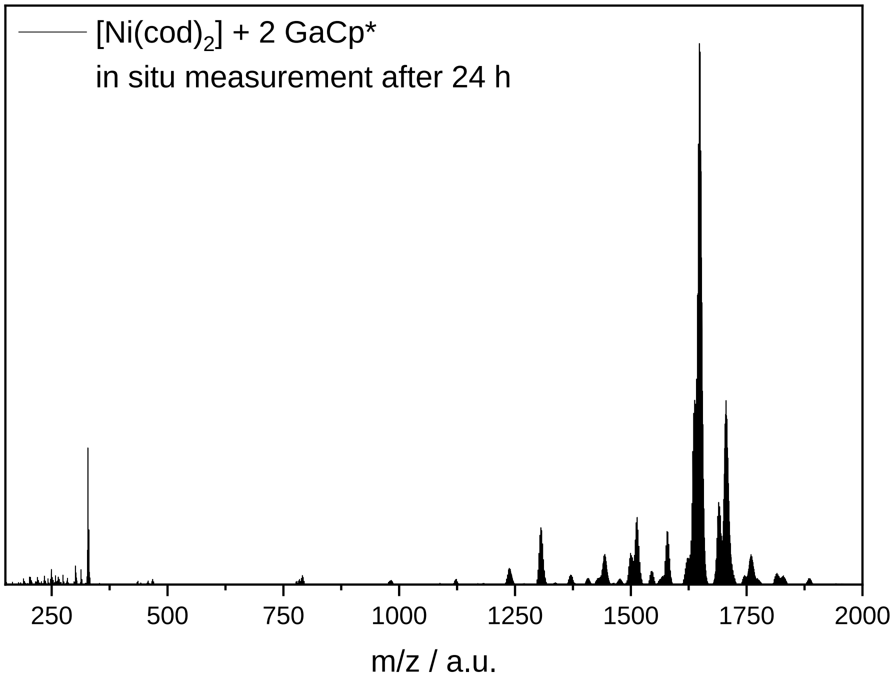

Figure S14. Full LIFDI mass spectra of the reaction mixture of 1.0 equiv. [Ni(cod)$_2$] and 2.0 equiv. GaCp* in toluene at 60 °C after 24 h (analog to the synthesis of **1A**) showing the similar signals compared to isolated crystals of **1A** (major component **3**) and some additional signals with lower and higher masses.

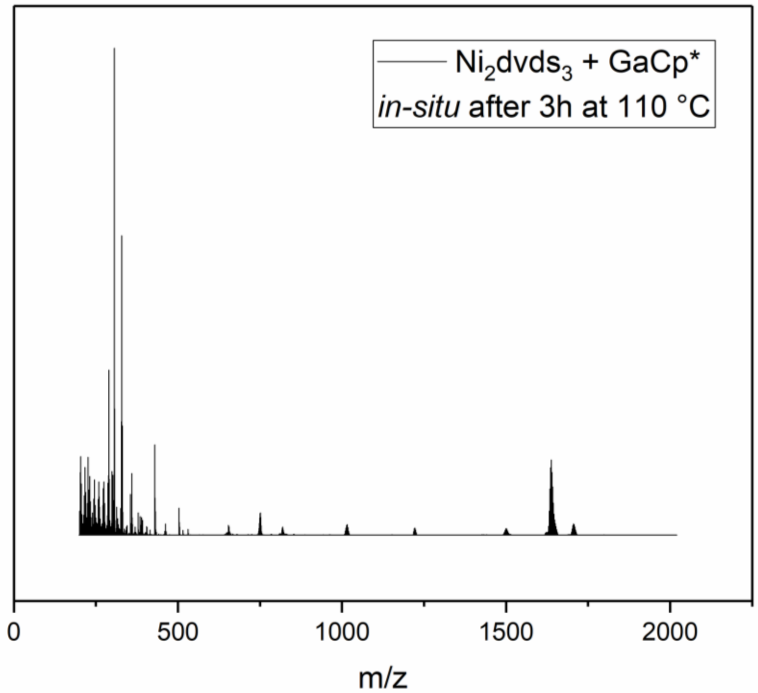

Figure S15. *In-situ* LIFDI-MS spectrum of the reaction of Ni_2dvds_3 and 2 eq GaCp* in toluene at 110 °C after 3 h. Similar signals compared to isolated crystals of **1B** (major component **2**) and some additional signals with lower masses are observed.

Labelling and fragmentation experiments

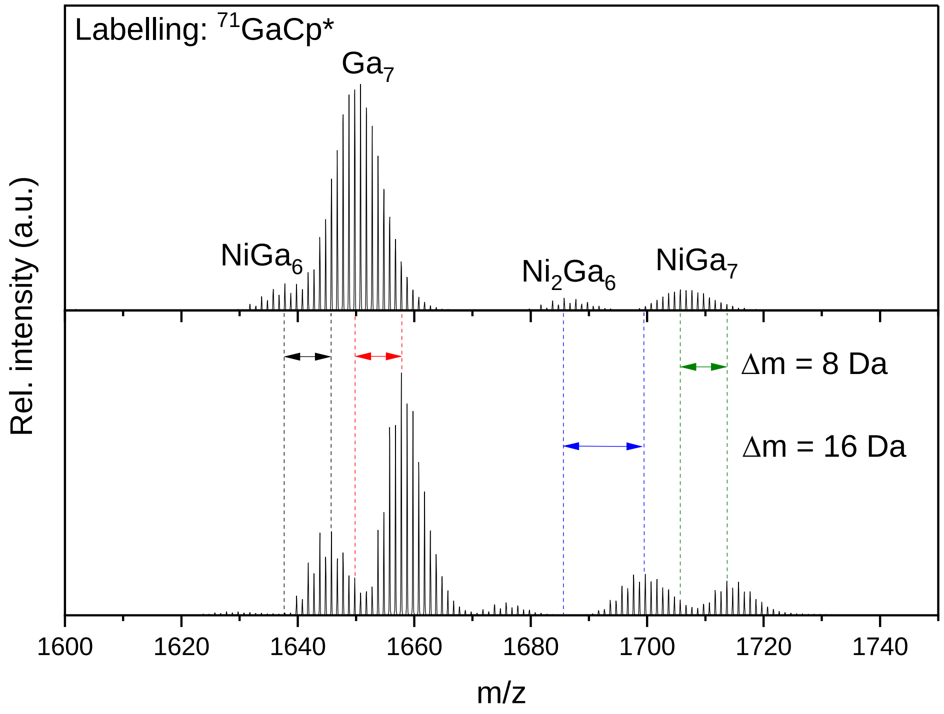

Figure S16. Cutout of the LIFDI MS spectrum of the labelling experiment ($Ni(cod)_2$ (1.0 eq.) and ^{71}GaCp* (1.0 eq.) in toluene at 60 °C for 24 h), showing a mass shift of *m/z* = 8 (top: LIFDI MS spectrum of GaCp* supported cluster ensemble with natural abundance, bottom: LIFDI MS spectrum of ^{71}Ga-labelled ensemble). **REMARK:** The Ni_2Ga_6 cluster shows a higher shift of 16 Da due to its low concentration and enhanced fragmentation of the molecular ion; nevertheless, the observed and calculated pattern of the ^{71}Ga labelled species fit well (s. Figure S17 below).

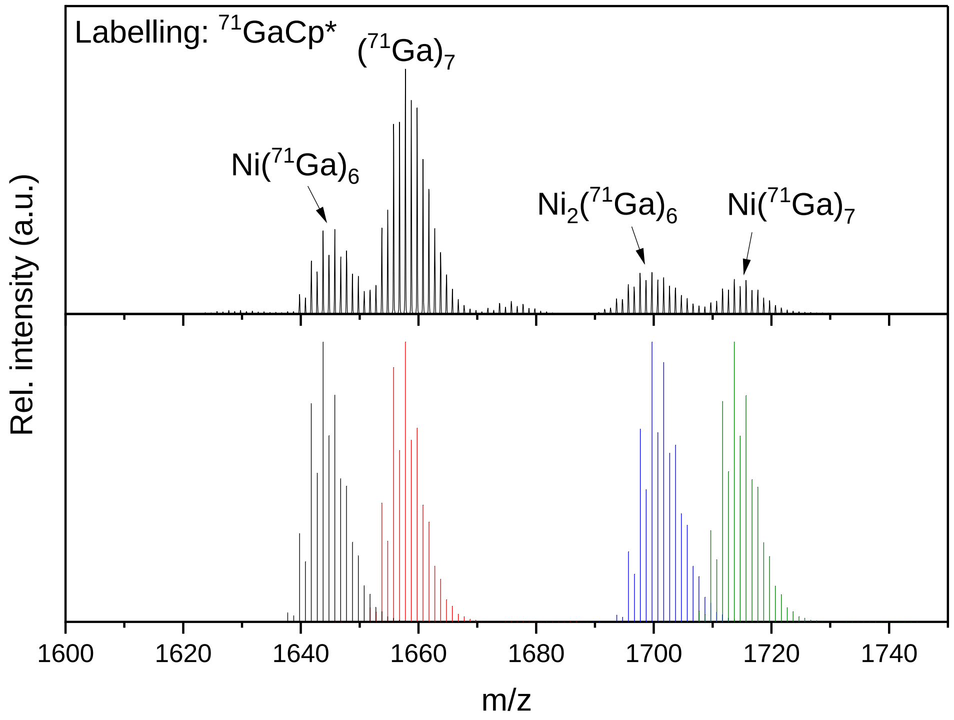

Figure S17. Comparison of measured (top) and calculated (bottom) isotopic patterns of ^{71}GaCp* labelled cluster species obtained from $Ni(cod)_2$ (1.0 eq.) and ^{71}GaCp* (1.0 eq.) in toluene at 60 °C after 24 h.

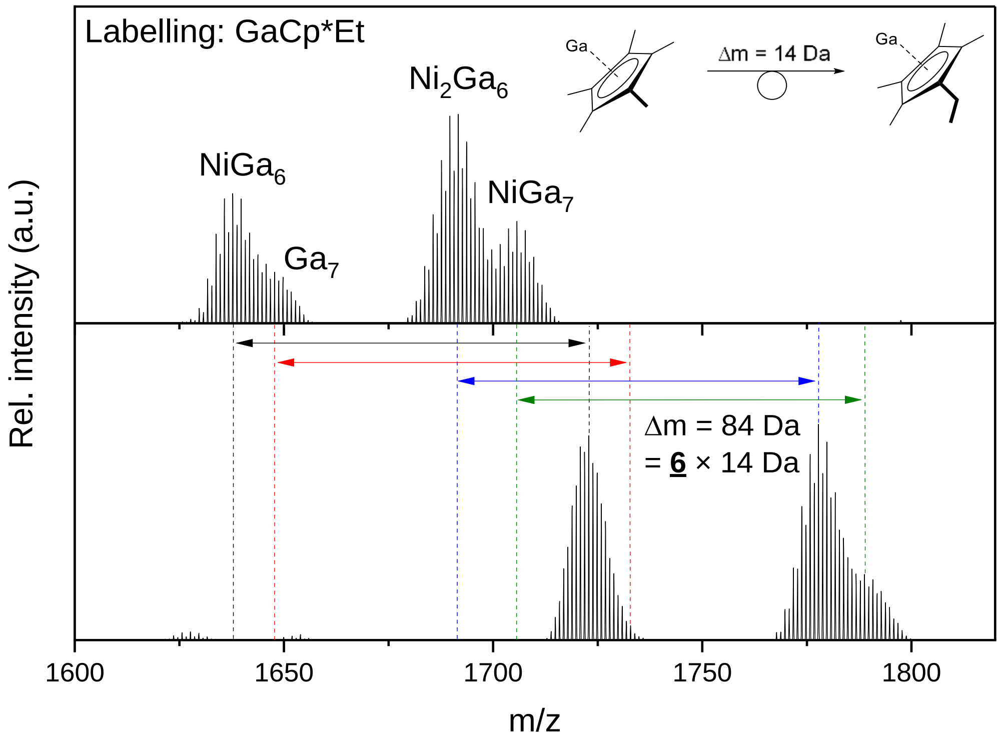

Figure S18. Comparison of Cp* (top) and Cp*Et supported cluster ensembles (bottom) generated from Ni(cdt) (1.0 eq.) and GaCp* (1.0 eq.) in toluene at 110 °C (4 h); all Cp*Et labelled cluster species show a mass shift of 84 Da indicating six Cp* units per cluster.

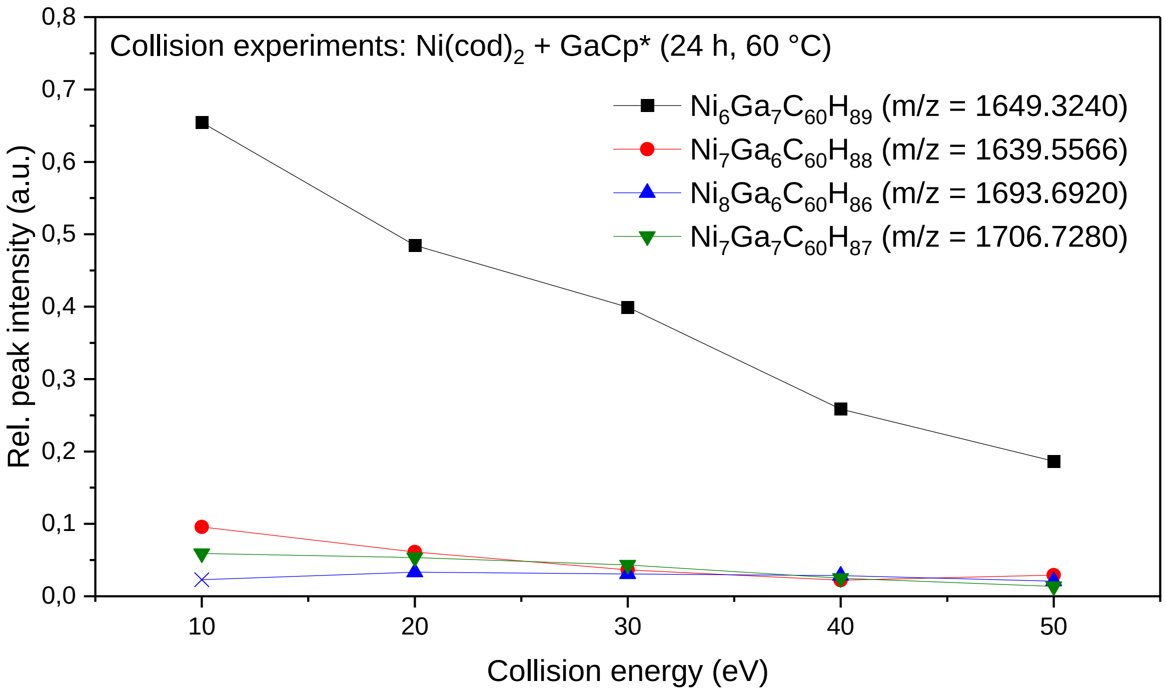

Figure S19. I vs. CE plots for molecular ions of the reaction mixture generated from $Ni(cod)_2$ (1.0 eq.) and GaCp* (1.0 eq.) in toluene at 60 °C after 24 h; outliers are highlighted with a cross. Sum formulas and m/z values refer to the highest intensity peak detected for the corresponding molecular ion.

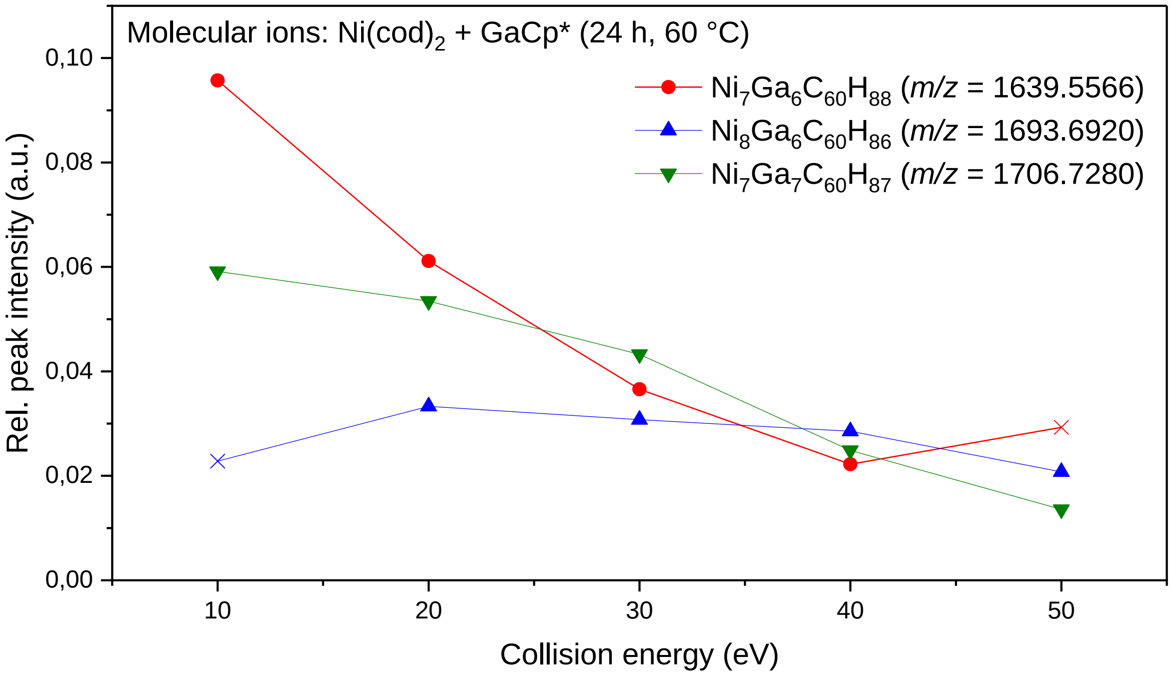

Figure S20. Cutout of the I vs. CE plots for the cluster species $Ni_7Ga_6(Cp^*)_6$, $Ni_8Ga_6(Cp^*)_6$ and $Ni_7Ga_7(Cp^*)_6$; outliers are highlighted with a cross. Sum formulas and m/z values refer to the highest intensity peak detected for the corresponding molecular ion.

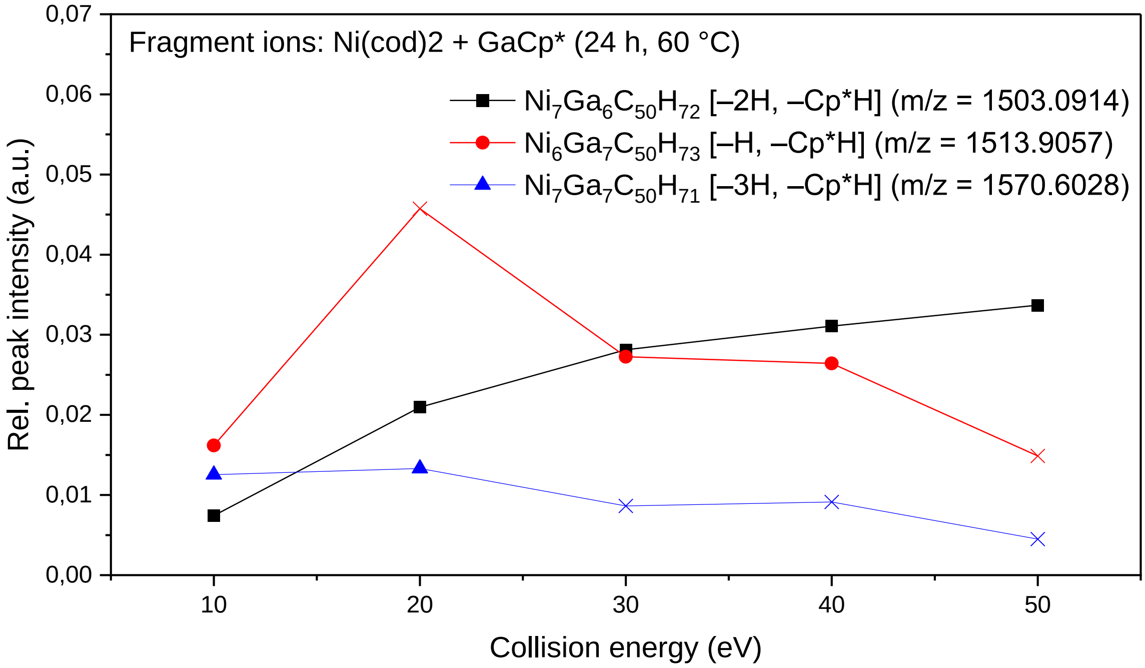

Figure S21. I vs. CE plots for fragment ions of the library generated from Ni(cod)2 and GaCp*; outliers highlighted with a cross most probably occur due to further fragmentation or decomposition of fragment ions. Due to the low abundance of the $Ni_8Ga_6(Cp^*)_6$ cluster in the cluster mixture, no fragment ions could be detected for this species.

Table S22: Integrals of both molecular and fragment ions of the cluster species generated from Ni(cod)2 and GaCp* relative to the overall integral of the spectrum (m/z = 300 to 2500). The fragment ion (– Cp*H) for $Ni_8Ga_6(Cp^*)_6$ was not observed due to the low abundance of the cluster within the whole ensemble.

**Cluster (fragment)**	**Collision energy (eV)**				
	**10**	**20**	**30**	**40**	**50**
**$Ni_6Ga_7C_{60}H_{89}$**	0.654465762	0.484569039	0.39933243	0.258541128	0.186585569
**$Ni_6Ga_7C_{50}H_{73}$** (– C_5Me_5H)	0.01618521	0.04572776	0.02725502	0.02642154	0.01488399
**$Ni_7Ga_6C_{60}H_{88}$**	0.09572775	0.061127733	0.036558998	0.022230692	0.029292605
**$Ni_7Ga_6C_{50}H_{72}$** (– C_5Me_5H)	0.00742046	0.02095377	0.02812307	0.03107572	0.0336989
**$Ni_8Ga_6C_{60}H_{86}$**	0.022815514	0.033316086	0.030737721	0.028555633	0.020761475
**$Ni_8Ga_6C_{50}H_{70}$** (– C_5Me_5H)	-	-	-	-	-
**$Ni_7Ga_7C_{60}H_{87}$**	0.059130255	0.05342917	0.04327831	0.024885336	0.013566397
**$Ni_7Ga_7C_{50}H_{71}$** (– C_5Me_5H)	0.01253219	0.01331351	0.00862967	0.00912603	0.00450173

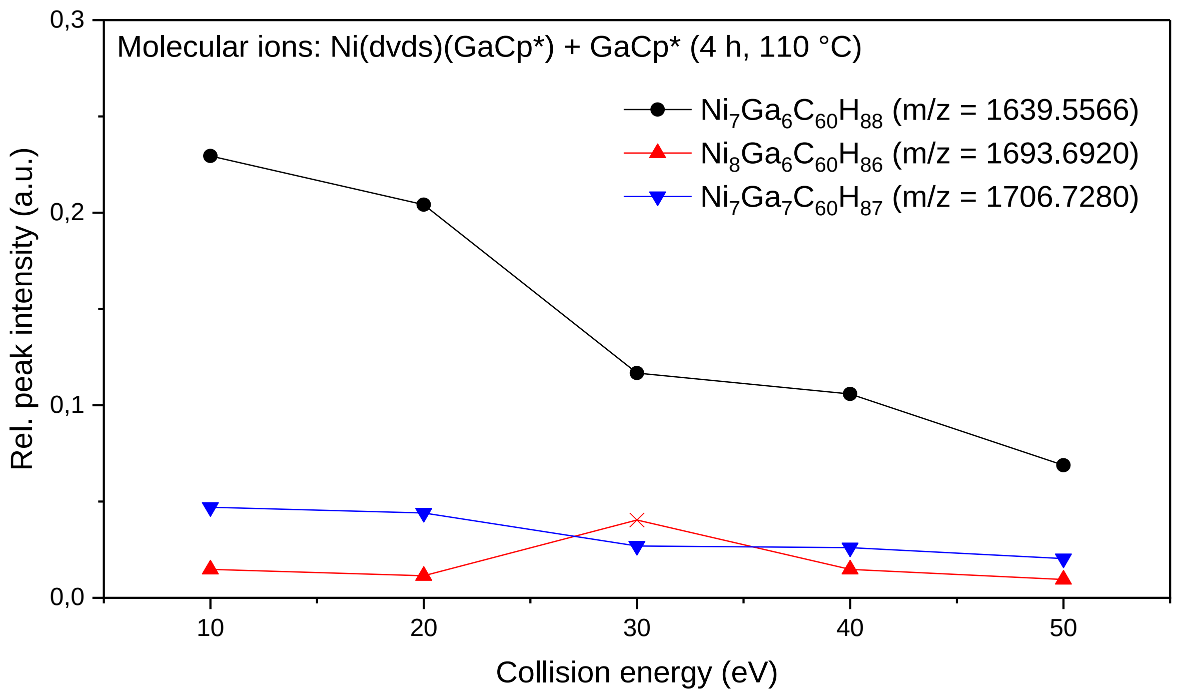

Figure S22. I vs. CE plots for molecular ions of the reaction mixture generated from Ni(dvds)(GaCp*) (1.0 eq.) and GaCp* (1.0 eq.) in toluene at 110 °C after 4 h; outliers are highlighted with a cross. Sum formulas and m/z values refer to the highest intensity peak detected for the corresponding molecular ion.

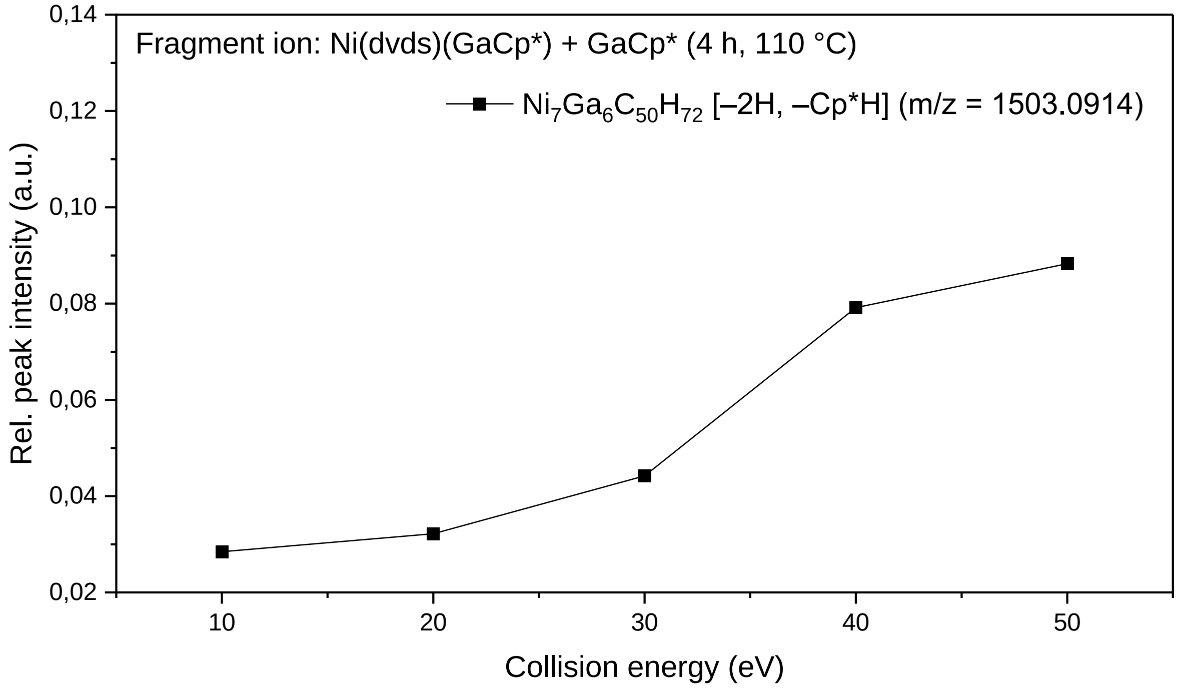

Figure S23. I vs. CE plots for the fragment ion $Ni_7Ga_6(Cp^*)_6^+$ of the library generated from Ni(dvds)(GaCp*) (1.0 eq.) and GaCp* (1.0 eq.) in toluene at 110 °C after 4 h. Due to the low abundance of other cluster species in this cluster mixture, no other fragment ions could be detected.

Table S23: Integrals of both molecular and fragment ions of the cluster species generated from Ni(dvds)(GaCp*) and GaCp* relative to the overall integral of the spectrum (m/z = 300 to 2500). The fragment ion (– Cp*H) for $Ni_8Ga_6(Cp^*)_6$ and $Ni_7Ga_7(Cp^*)_6$ were not observed due to the low abundance of the clusters within the whole ensemble.

**Cluster (fragment)**	**Collision energy (eV)**				
	**10**	**20**	**30**	**40**	**50**
**$Ni_7Ga_6C_{60}H_{88}$**	0.229476339	0.204154913	0.116725806	0.105824416	0.068871334
**$Ni_7Ga_6C_{50}H_{72}$** (– C_5Me_5H)	0.028458224	0.032193253	0.044209176	0.079159581	0.088270957
**$Ni_8Ga_6C_{60}H_{86}$**	0.014802333	0.011424329	0.040398352	0.014812056	0.009540365
**$Ni_8Ga_6C_{50}H_{70}$** (– C_5Me_5H)	-	-	-	-	-
**$Ni_7Ga_7C_{60}H_{87}$**	0.047053752	0.044046463	0.026924592	0.026041055	0.02039919
**$Ni_7Ga_7C_{50}H_{71}$** (– C_5Me_5H)	-	-	-	-	-

SCXRD analysis

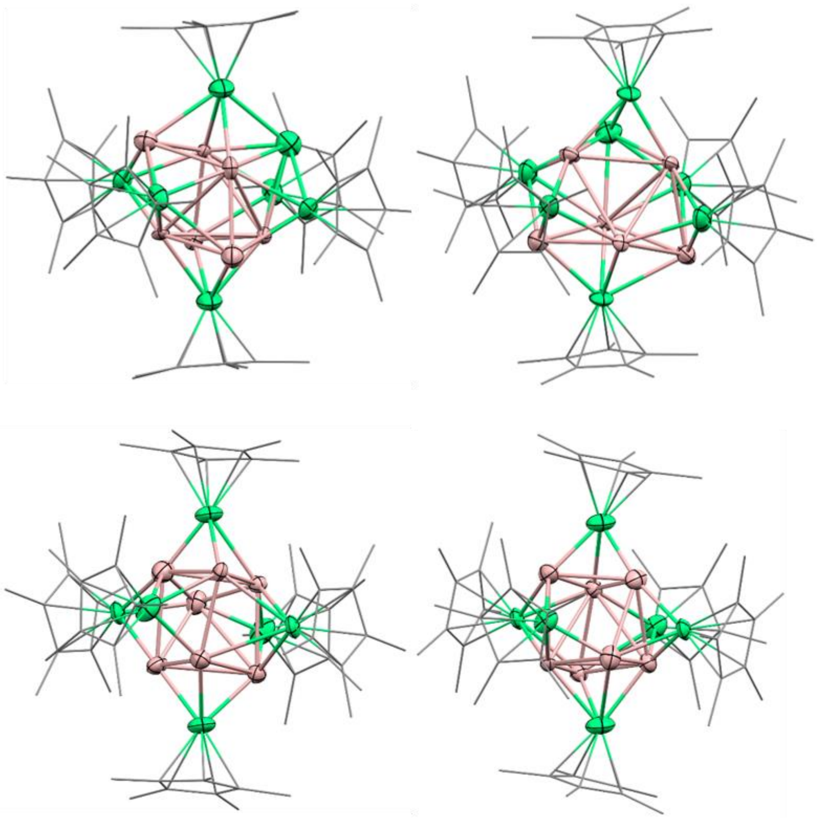

Figure S24. Optimized structures obtained by SC-XRD measurements.

Crystal data (CCDC deposition number: 2077846)

$(C_{60}H_{90}Ga_{6.66}Ni_{6.61})\cdot 2(C_9H_{12})$	$F(000)$ = 973
M_r = 1904.01	
Triclinic, *P*	D_x = 1.655 Mg m^{-3}
Hall symbol: P-1	Melting point: ? K
a = 12.6698 (7) Å	Mo *Kα* radiation, λ = 0.71073 Å
b = 12.7434 (7) Å	Cell parameters from 9932 reflections
c = 13.9656 (8) Å	θ = 2.2–25.7°
α = 88.476 (2)°	μ = 3.93 mm^{-1}
β = 69.582 (2)°	*T* = 100 K
γ = 65.805 (2)°	Fragment, black
V = 1909.59 (19) Å^3	0.45 × 0.30 × 0.27 mm
Z = 1	

Data collection

Bruker Photon CMOS diffractometer	6995 independent reflections
Radiation source: IMS microsource	6070 reflections with $I > 2\sigma(I)$
Helios optic monochromator	R_{int} = 0.028
Detector resolution: 16 pixels mm^{-1}	θ_{max} = 25.4°, θ_{min} = 2.2°
phi– and ω–rotation scans	h = −15 15
Absorption correction: multi-scan *SADABS* 2016/2, Bruker	k = −15 15
T_{min} = 0.503, T_{max} = 0.745	l = −16 16
50781 measured reflections	

Refinement

Refinement on F^2	Secondary atom site location: difference Fourier map
Least-squares matrix: full	Hydrogen site location: inferred from neighbouring sites
$R[F^2 > 2\sigma(F^2)]$ = 0.039	H-atom parameters constrained
$wR(F^2)$ = 0.097	W = $1/[\Sigma^2(FO^2) + (0.040P)^2 + 3.3861P]$ WHERE $P = (FO^2 + 2FC^2)/3$
S = 1.04	$(\Delta/\sigma)_{max}$ = 0.004
6995 reflections	$\Delta\rho_{max}$ = 0.55 e Å^{-3}
914 parameters	$\Delta\rho_{min}$ = −0.65 e Å^{-3}
1633 restraints	Extinction correction: none
0 constraints	Extinction coefficient: -
Primary atom site location: iterative	

PXRD analysis

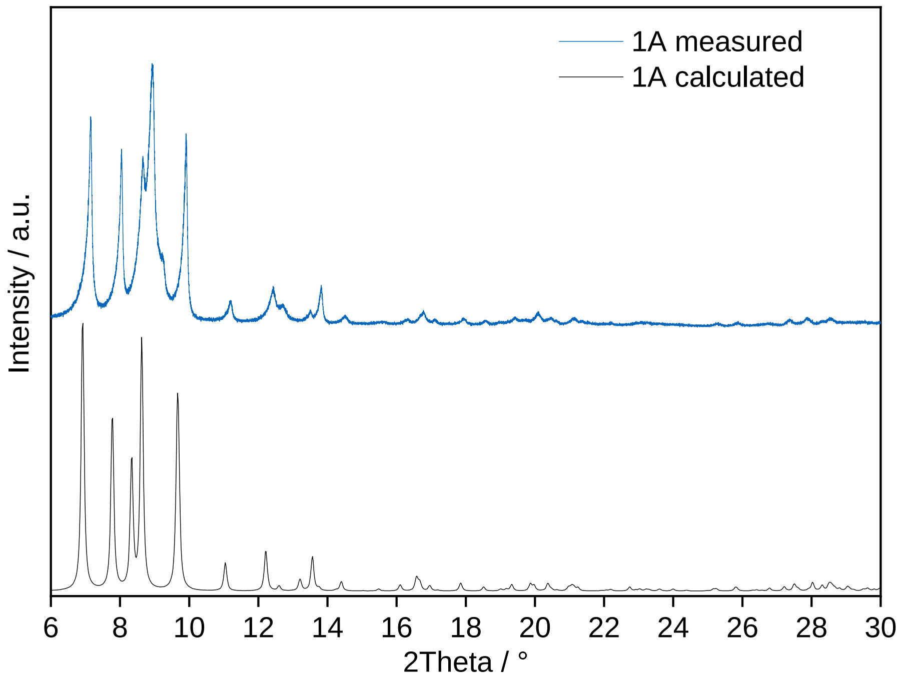

Figure S25. PXRD measurement of **1A** (major component **3**) and comparison with calculated pattern out of SXRD measurements.

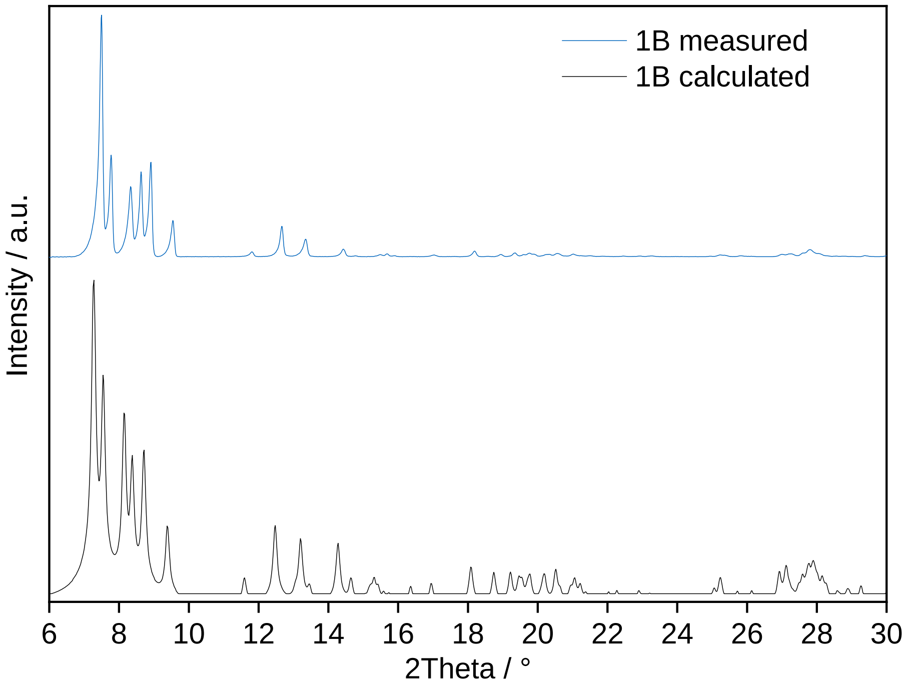

Figure S26. PXRD measurement of **1B** (major component 2) and comparison with calculated pattern out of SXRD measurements.

XPS measurements

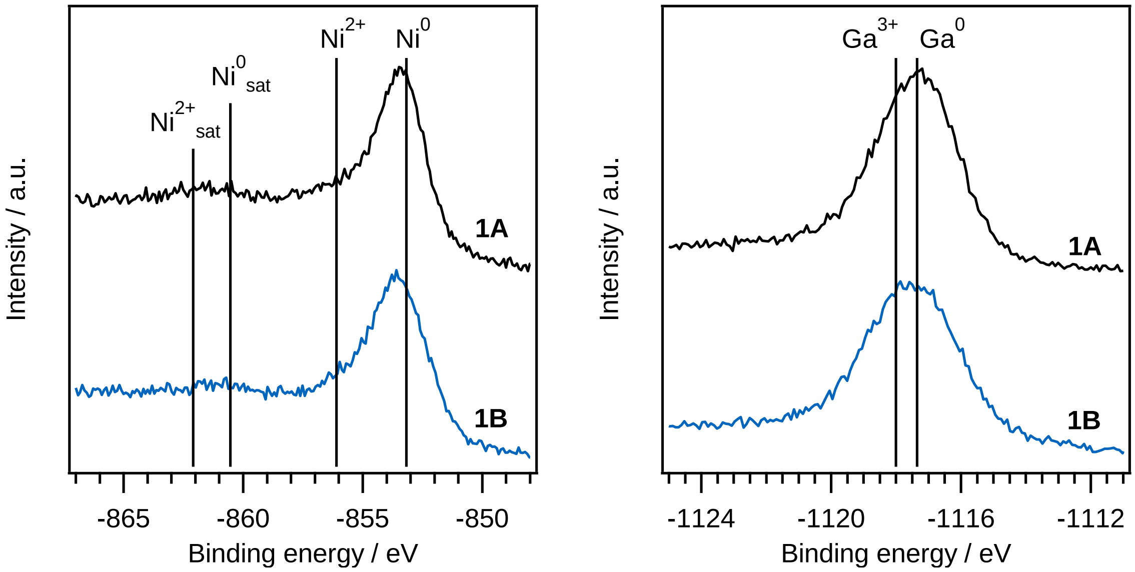

Figure S27. Ni $2p_{3/2}$ (left) and Ga $2p_{3/2}$ (right) photoemission spectra of **1A** (major component **3**) and **1B** (major component 2) evidence the presence of both metals predominately in their metallic state. For comparison, lines pinpoint the Ni $2p_{3/2}$ binding energy of Ni^0 and Ni^{2+} species (main line and satellite) as well as the Ga $2p_{3/2}$ binding energy of Ga^0 and Ga^{3+} species, respectively. As the binding energy of metallic and oxidic species in the cluster components **1A** and **1B** might vary from the one obtained for bulk materials we determined the binding energy for the oxidized species after oxidizing the clusters in air. The metallic reference was obtained by electron-induced reduction upon Ar^+ ion sputtering. The oxidation states were confirmed considering the peak position and shape of the Ni LMM and Ga LMM Auger spectra, respectively. Quantitative analysis of the Ni $2p_{3/2}$ and Ga $2p_{3/2}$ spectra reveals a molar Ni/Ga ratio of 1.1 (**1A**) and 1.0 (**1B**).

Elemental Analysis

Table S24: AAS analysis of **1A** (major component **3**) and **1B** (major component **2**) and theoretical values of various cluster compounds.

	**%C**	**%H**	**%Ni**	**%Ga**	**Ni/Ga ratio**
1A	42.6	5.5	27.4	24.3	1.12
1B	45.0	5.6	25.1	24.2	1.04
$[Ni_8Ga_6](Cp^*)_6$	42.3	5.7	27.5	24.5	1.12
$[Ni_7Ga_6](Cp^*)_6$	43.7	5.8	24.9	25.4	0.98
$[Ni_7Ga_7](Cp^*)_6$	42.0	5.6	23.9	28.4	0.84
$[Ni_6Ga_7](Cp^*)_6$	43.5	5.8	21.2	29.4	0.72
$[Ni_9Ga_5](Cp^*)_6$	42.7	5.4	31.3	20.7	1.51

SQUID and EPR measurements

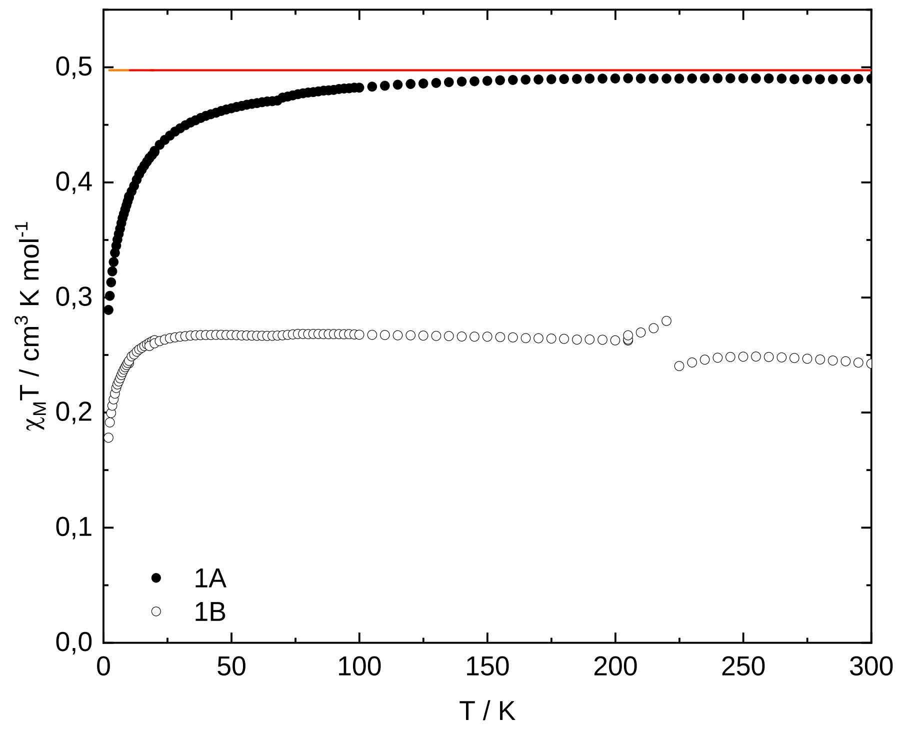

Figure S28. χ_MT of **1A** (major component **3**; closed circles) and **1B** (major component **2**; open squares) detected by SQUID measurements plotted against the temperature T. The red line indicates an isolated spin ½ with g = 2.30.

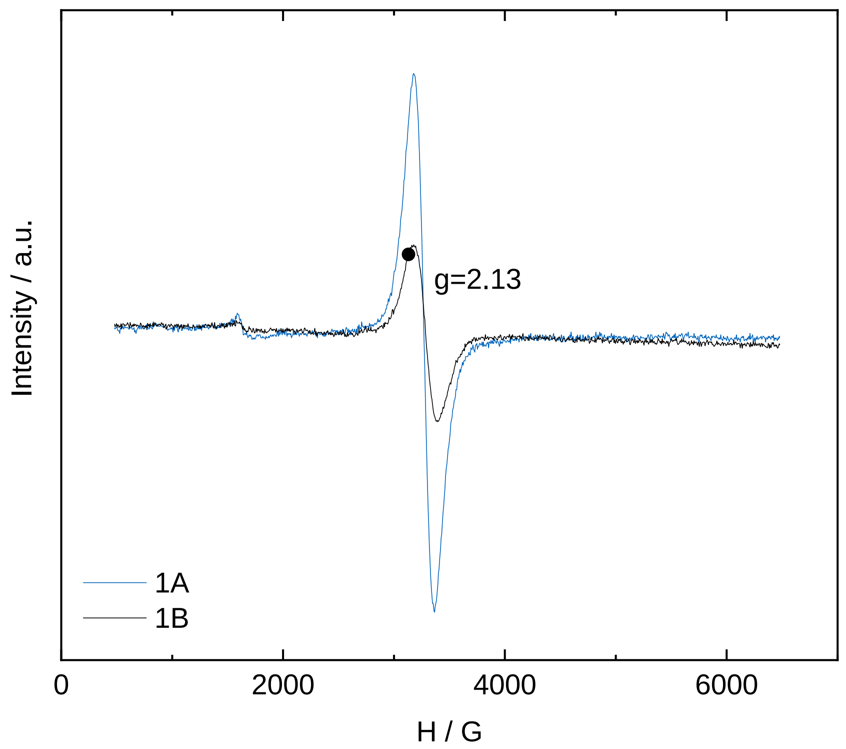

Figure S29: EPR spectra of **1A** (major component **3**; blue) and **1B** (major component **2**; black) measured at room temperature.

Procedures and Strategies for the Atomic Structure Generation

Here, we propose a systematic approach to study of the M_{13} and M_{14} clusters protected by $(C_5Me_5)_6$ ligands. Figure S30 summarizes the strategy and procedures employed to generate the atomic structure configurations. As we show in the Figure, initially the study is divided on two pathways. The right side of the scheme was tentatively performed for the naked clusters based on the XRD data. Furthermore, on the left of the scheme, a pure theoretical approach based on a previous work from our group, in which the transition metal (TM) clusters with 215 atoms were studied. These paper shows a rich conformational set for unary clusters that was used to enrich our structural set.

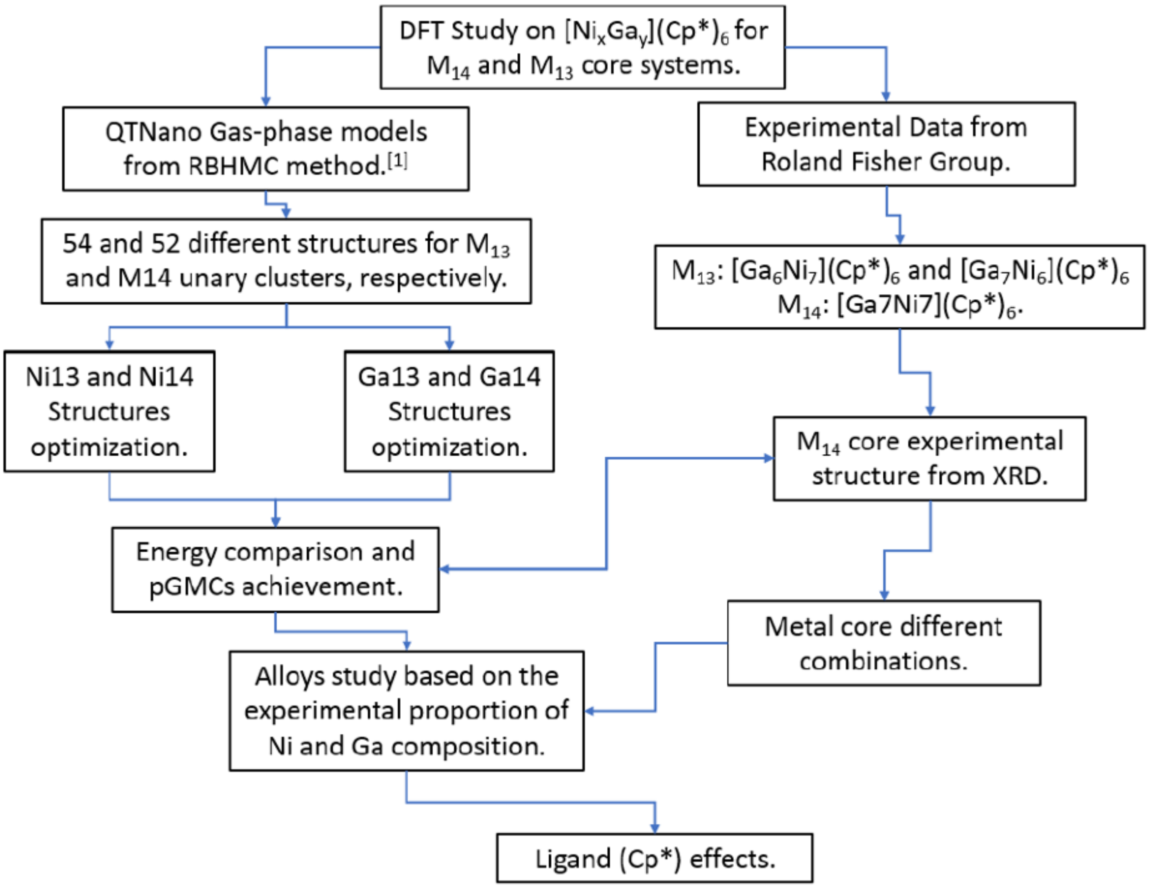

Figure S30. Systematic approach to generate atomic structure configurations for the M13 and M14 clusters.

Structural Analyses of the Unary and Binary Clusters

To characterize the most important structural parameters of the unary and binary clusters, namely, coordination number and bond lengths, we employed the effective coordination concept (ECC), which yields the following parameters: (i) weighted bond lengths, d^i_{av}, and effective coordination number, ECN^i, in number of nearest neighbor (NNN), for each atom, *i*, within the cluster. Those values are obtained using a self-consistent approach based on exponential decay functions. The ECC considers that every atom *i* is surrounded by atoms *j* at different differences, and using an exponential decay function, a weight is calculated for each *i j* distance. As expected, the closest atoms have a larger weight and the more distant ones have a smaller weight. Thus, ECN^i is obtained by the following equation:

$$ECN^i = \sum_j exp\left[1 - \left(\frac{d_{ij}}{d^i_{av}}\right)^6\right]$$

where d_{ij} is the distance between atom *i* and *j*, while d^i_{av} is obtained by the following equation:

$$d^{i,new}_{av} = \frac{\sum_j d_{ij} exp\left[1 - \left(\frac{d_{ij}}{d^{i,old}_{av}}\right)^6\right]}{\sum_j exp\left[1 - \left(\frac{d_{ij}}{d^{i,old}_{av}}\right)^6\right]}$$

d^i_{av} is obtained self-consistently, i.e., $\left|d^{i,new}_{av} - d^{i,old}_{av} < 0.00010\right|$. The smallest bond length between the atom i and all j atoms, di min, is used as the initial value for d^i_{av}. The final values of d^i_{av} are obtained within 3-4 iterations, which are then used to calculated ECN_i as indicated above, which is obtained by the sum of all weights and does not have to be an integer value. The average results, ECN_{av} and d_{av}, are obtained by the following equations:

$$ECN_{av} = \frac{1}{N}\sum_{i=1}^{N} ECN^i$$

$$d_{av} = \frac{1}{N}\sum_{i=1}^{N} d^i_{av}$$

where N is the total number of atoms in the cluster. This approach is suitable for systems with lower symmetry, which is the case of systems addressed in this study.

Atomic Structure of the Unprotected Unary M13 and M14 Clusters

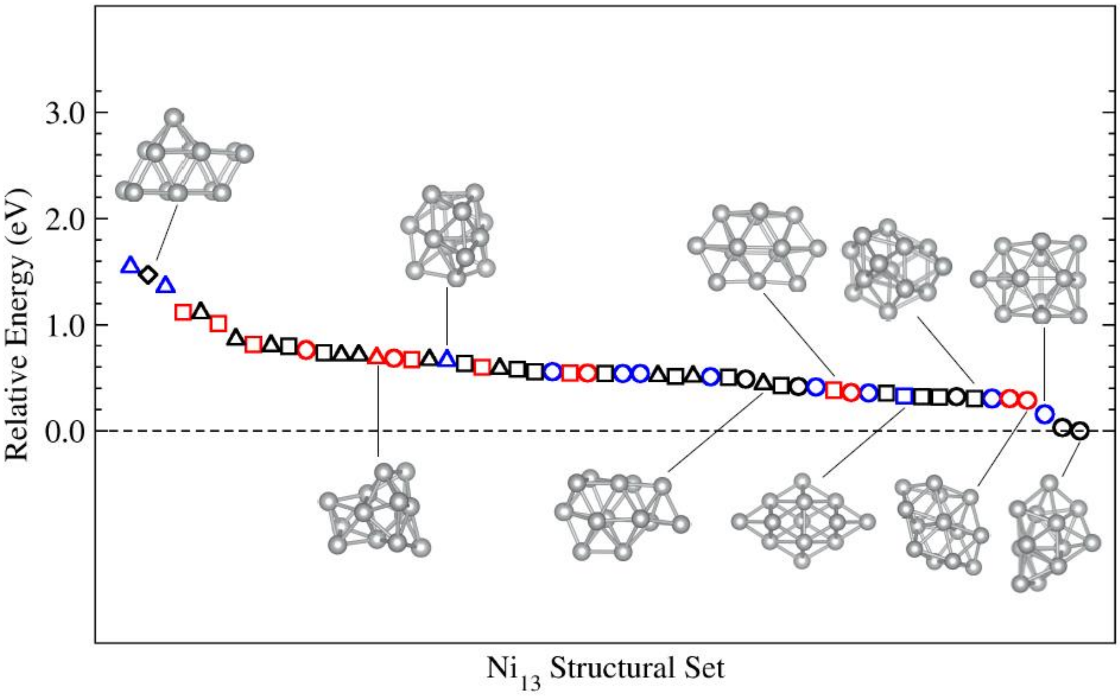

Figure S31. Atomic structure configurations optimized for the Ni_{13} clusters. Using clustering algorithms all optimized structures were separated into 10 groups, and the lowest energy configuration of each group are indicated within the figure.

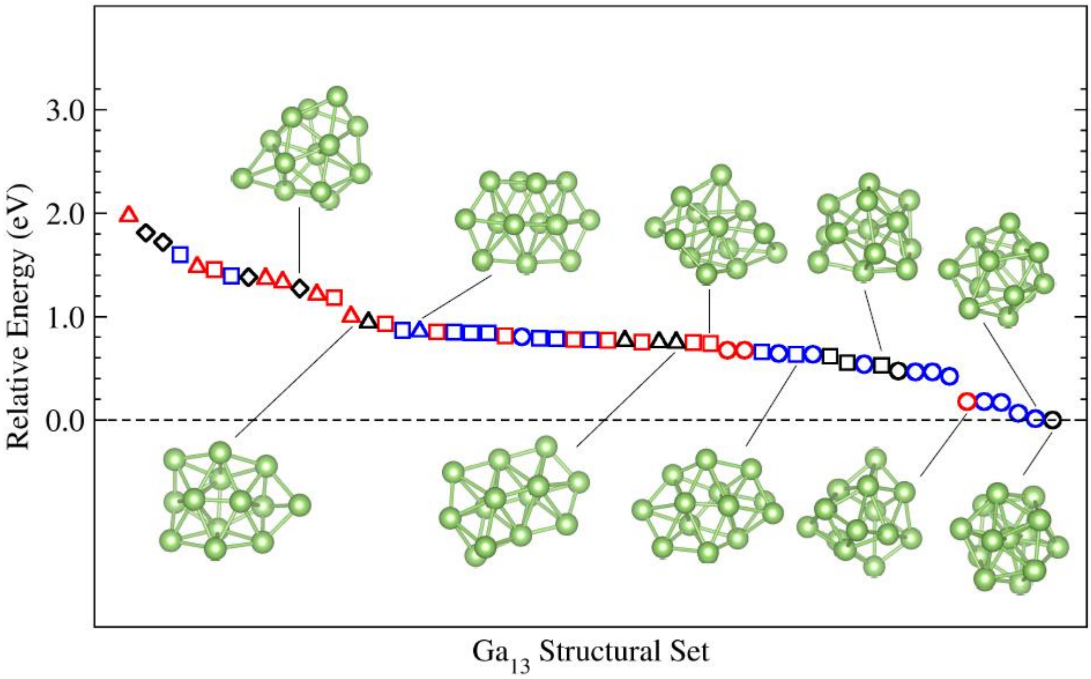

Figure S32. Atomic structure configurations optimized for the Ga_{13} clusters. Using clustering algorithms all optimized structures were separated into 10 groups, and the lowest energy configuration of each group are indicated within the figure.

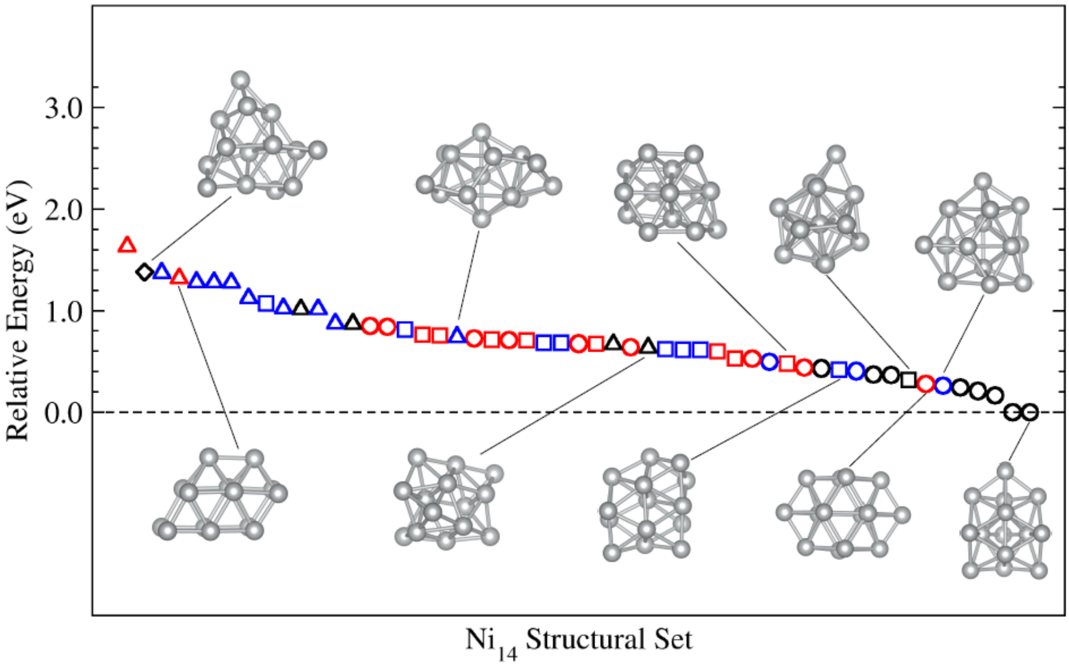

Figure S33. Atomic structure configurations optimized for the Ni_{14} clusters. Using clustering algorithms all optimized structures were separated into 10 groups, and the lowest energy configuration of each group are indicated within the figure.

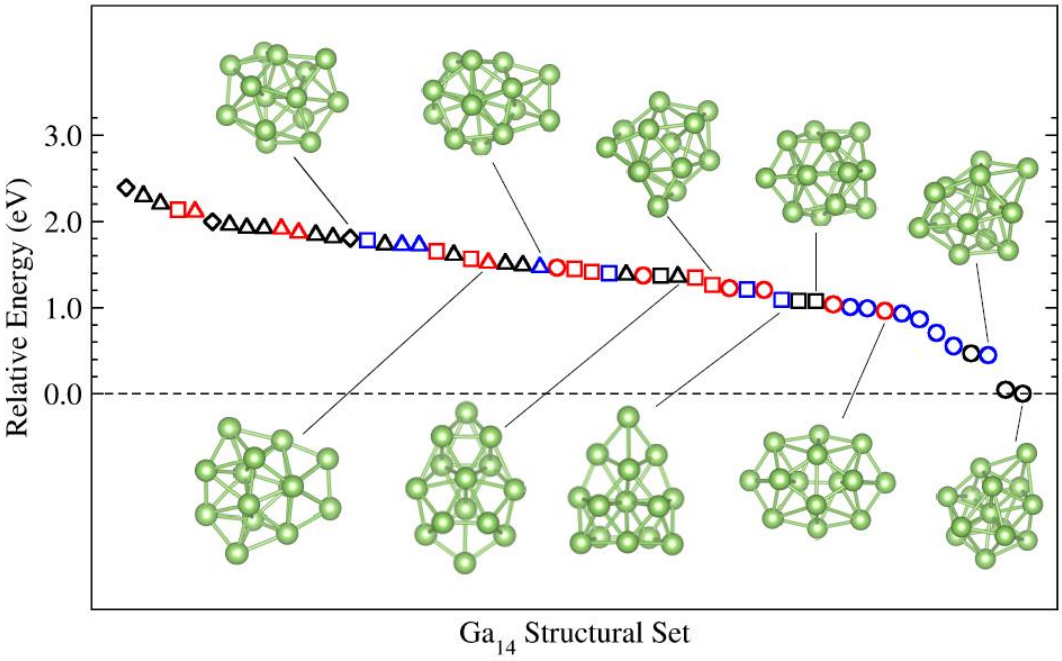

Figure S34. Atomic structure configurations optimized for the Ni_{13} clusters. Using clustering algorithms all optimized structures were separated into 10 groups, and the lowest energy configuration of each group are indicated within the figure.

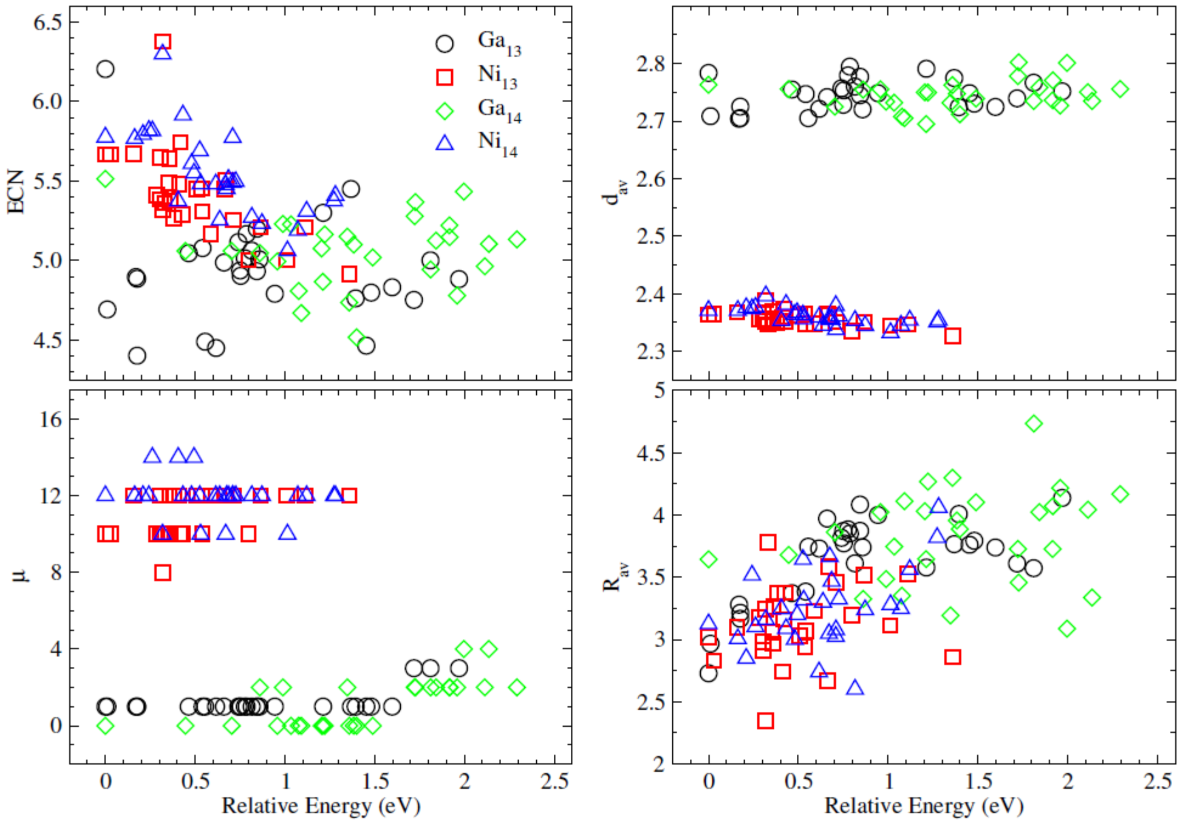

Figure S35. Structural parameters for the Ni_{13}, Ga_{13}, Ni_{14}, and Ga_{14} clusters: average effective coordination number, ECN, in number of nearest neighbor (NNN), average weighted bond length, d_{av}, in Å, average cluster radius, R_{av}, in Å, and dipole moment, μ, in Debye.

Unprotected Binary M_{13} and M_{14} Clusters

Structure Frame from XRD Results

Figure S36 indicates the structure for the M_{14} cluster obtained from the experimental XRD results without the indication of the ligands. Thus, based on the geometric analysis of the structure, we can provide the following observations: (i) the atoms indicated in light blue are near to the geometric center, and are called first shell, while the atoms in light pink belong to the second shell of atoms. (ii) the atoms indicated by green indicates the atoms in the equatorial vertices, while the remaining the dark brown atoms defined the axial vertices. Although the present structure framework is provided, the correct positions of the Ni and Ga was confirmed by density functional theory calculations by assuming all possible sites as occupied by the Ni or Ga atoms for a given composition. Thus, using an enumeration process, and analysis of the similarity among the configurations to remove similar structures (modified Euclidean metrics analysis), we could obtain and identify the lowest energy configurations for the protected clusters. Similar procedure was employed for M_{13} clusters.

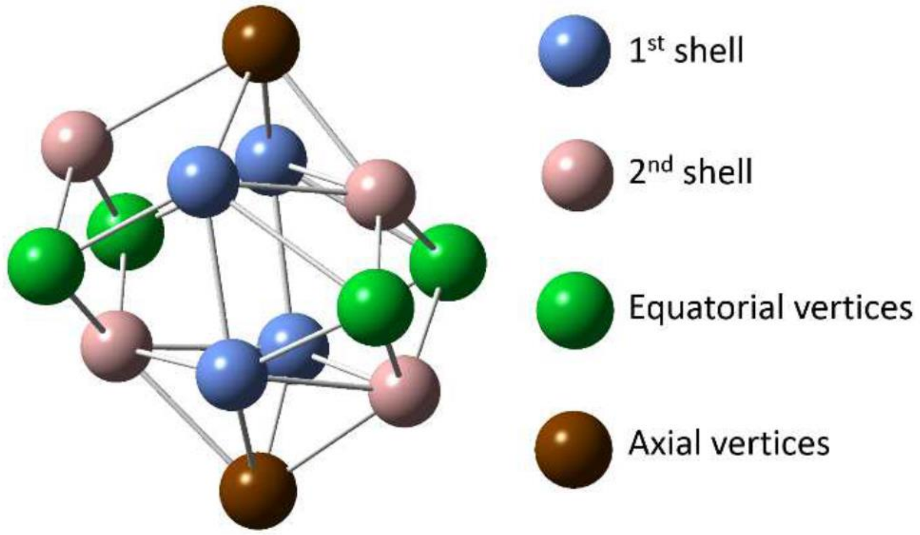

Figure S36. Atomic structure of the M_{14} cluster based on the SCXRD results.

Ni_7Ga_7

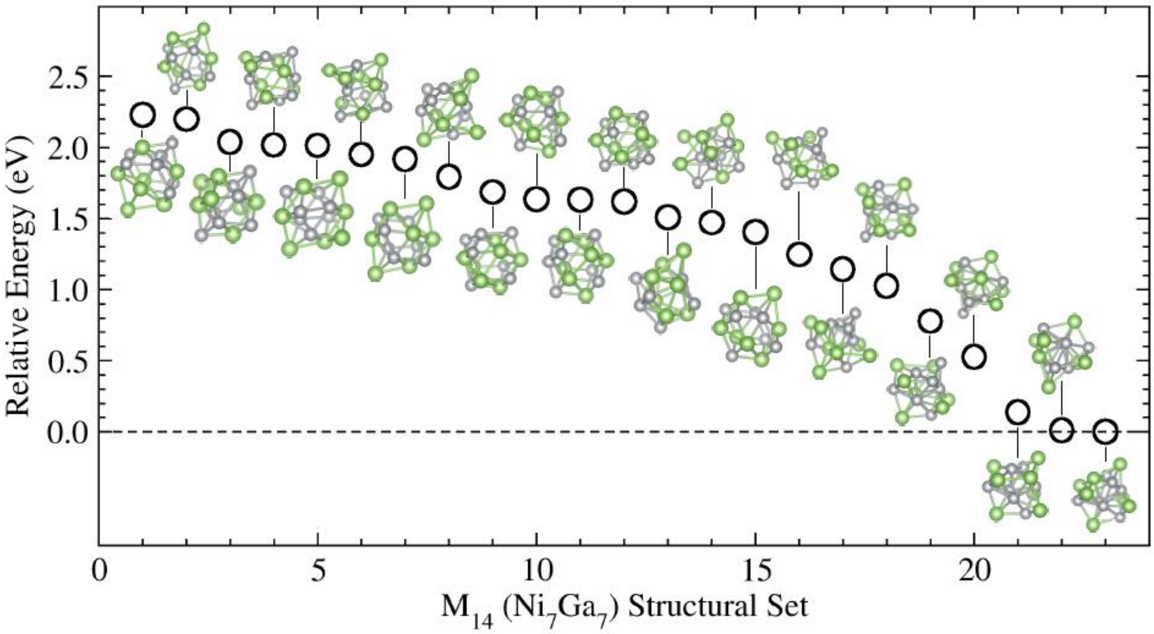

Figure S37. Structural set for the unprotected Ni_7Ga_7 clusters. The magnetic moment is equal to 1 for the major part of the structures, except for the structure 21, that is 1:25 eV above lowest energy configuration for these systems. In the lowest energy configuration, the Ni atoms preferential sites are in the inner sites of the clusters, and these trends is observed on the systems 18, 14, 23 and 2, that shows relative total energies smaller than 1 eV from the lowest energy configuration. The excess energy was further calculated, resulting to negative values for all the systems, that represents a stabilization of the alloy clusters in comparison to the unary Ni_{14} and Ga_{14} clusters.

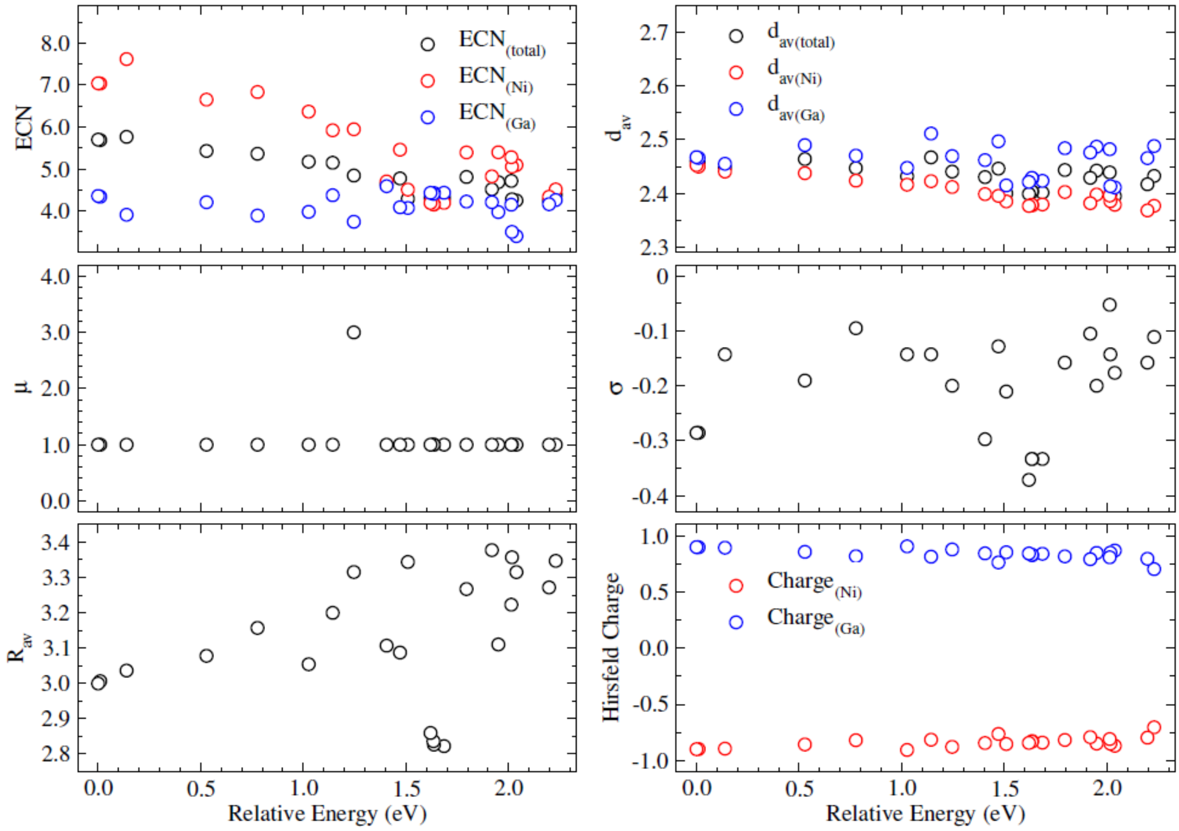

Figure S38. Geometrical and electronic analyses for the unprotected Ni_7Ga_7 clusters: average effective coordination number, ECN, in number of nearest neighbor (NNN), average weighted bond length, *dav*, in Å, average cluster radius, *Rav*, in Å, and dipole moment, *μ*, in Debye, chemical ordering parameter, *σ*, and effective Hirsfeld charge, *e*.

Ni_6Ga_7

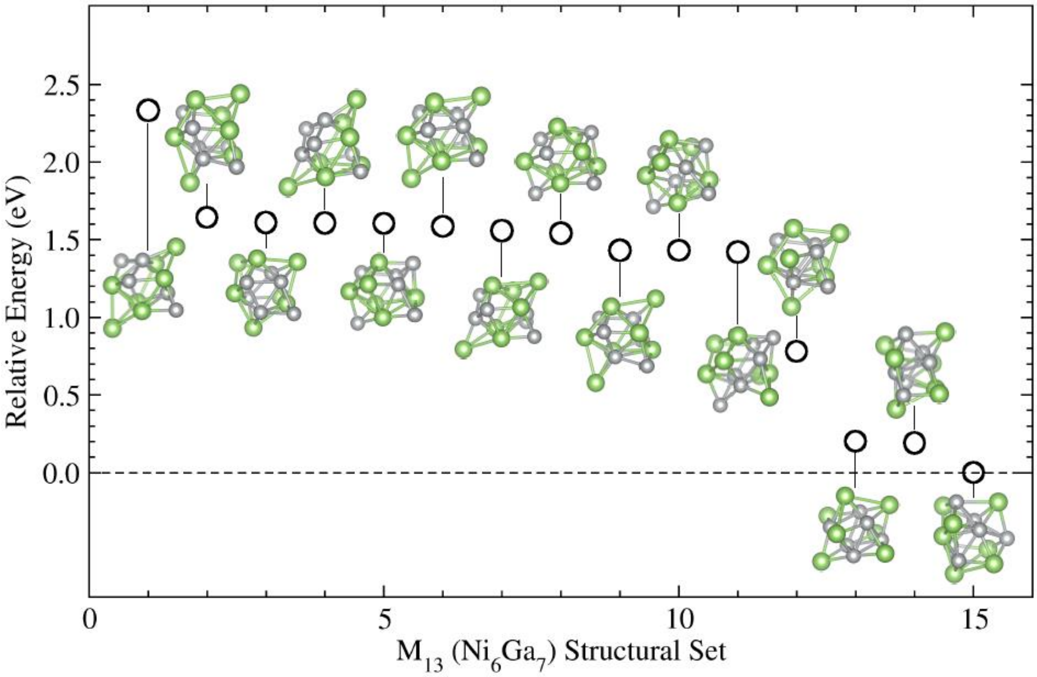

Figure S39. Structural set for Ni_6Ga_7 naked clusters.

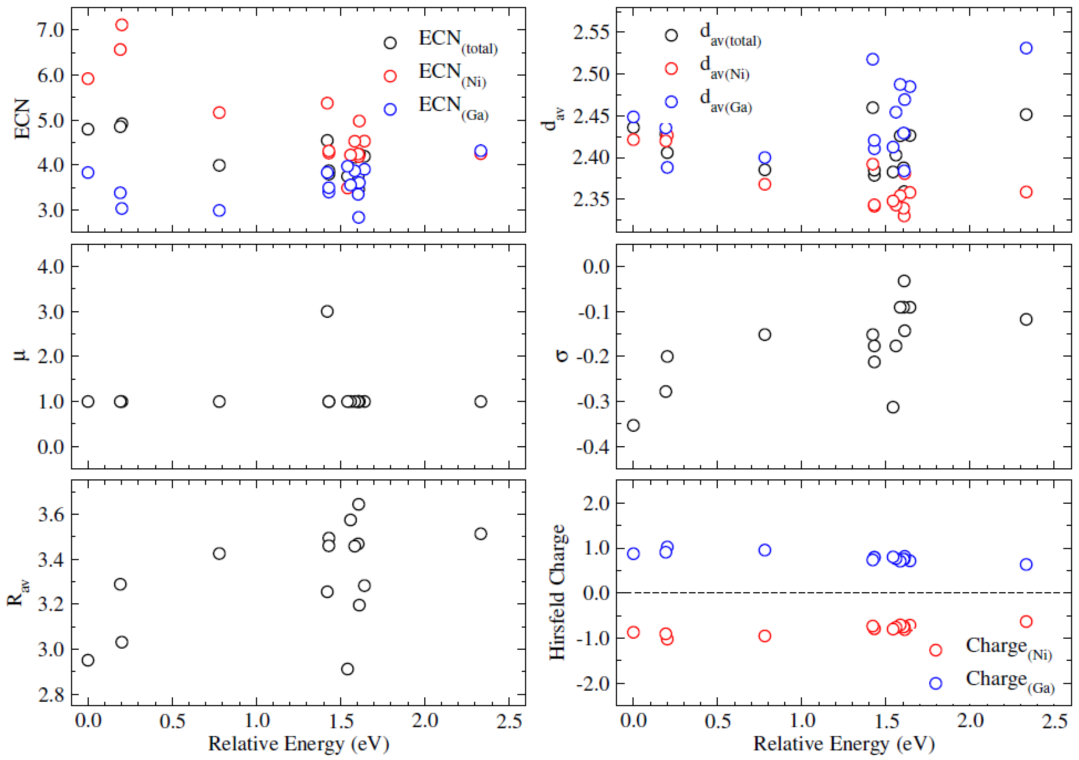

Figure S40. Geometrical and electronic analyses for the unprotected Ni6Ga7 clusters: average effective coordination number, ECN, in number of nearest neighbor (NNN), average weighted bond length, *dav*, in Å, average cluster radius, *Rav*, in Å, and dipole moment, *μ*, in Debye, chemical ordering parameter, *σ*, and effective Hirsfeld charge, *e*.

Ni_7Ga_6

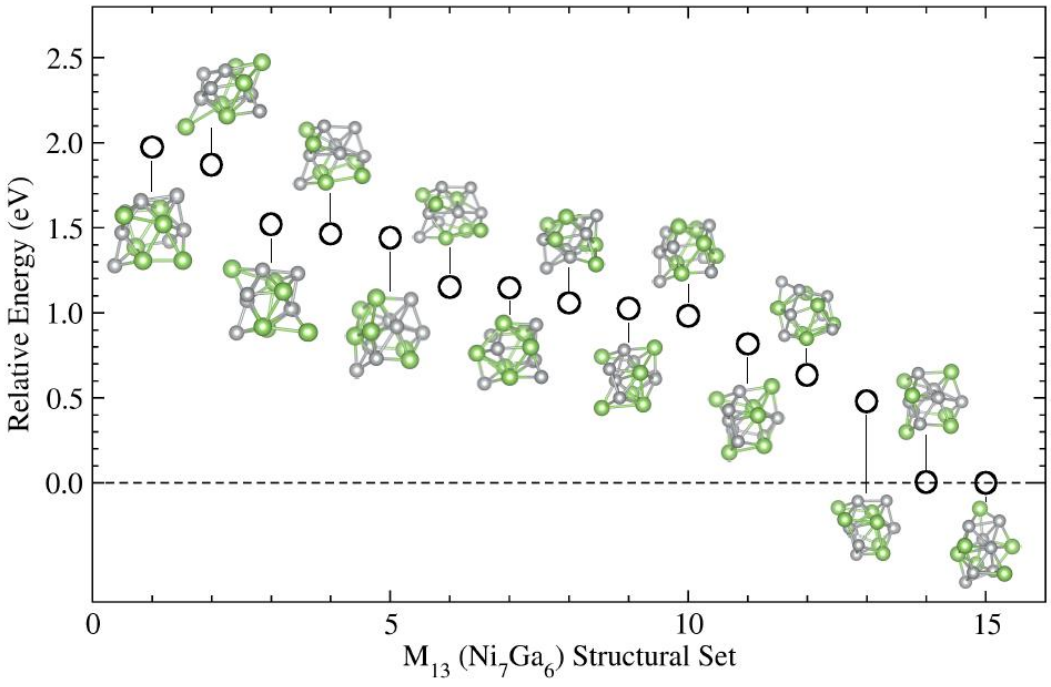

Figure S41. Structural set for Ni_7Ga_6 naked clusters.

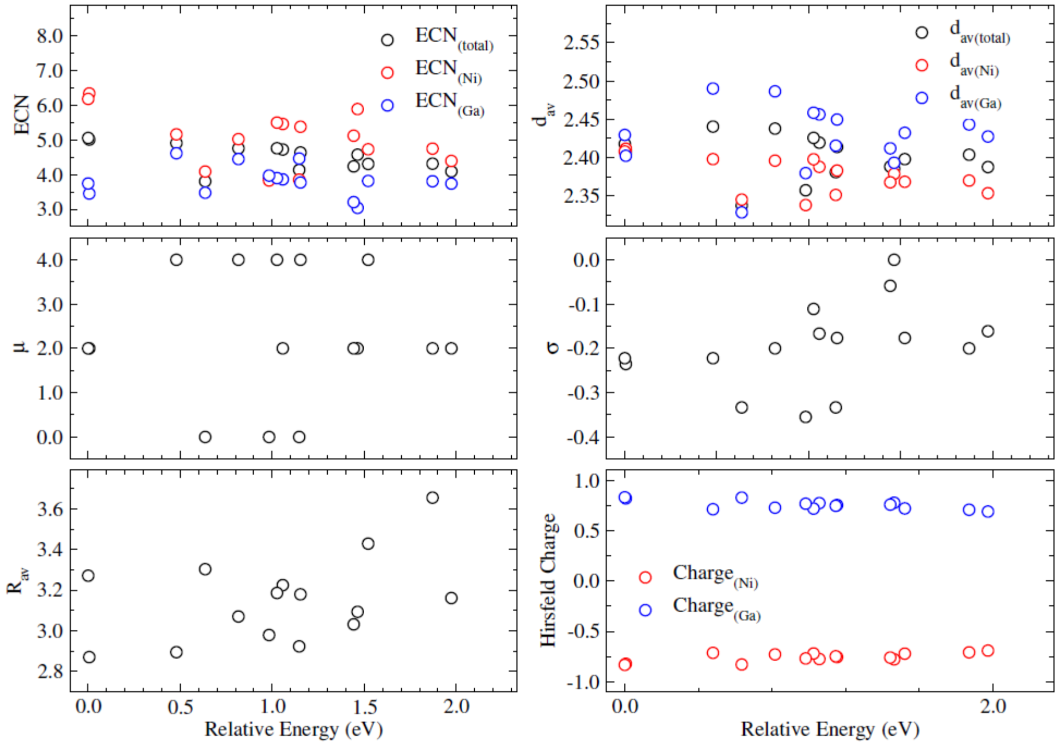

Figure S42. Geometrical and electronic analyses for the unprotected Ni7Ga6 clusters: average effective coordination number, ECN, in number of nearest neighbor (NNN), average weighted bond length, *dav*, in Å, average cluster radius, *Rav*, in Å, and dipole moment, *μ*, in Debye, chemical ordering parameter, *σ*, and effective Hirsfeld charge, *e*.

Protected Binary M_{13} and M_{14} Clusters

Ni_7Ga_7

Using the previous core structure separated in 4 different regions, as shown in Figure S36, were selected 10 different systems for the Cp* coordination study. We have inserted the Cp* ligands on the 6 vertices of the molecule, as showed on Figure S45. The relative energies were calculated considering the lowest energy's isomer as the referential (zero energy). If we compare to the previous results, on the naked clusters, the preferential sites for the Ni atoms migrate from the inner to the surfaces sites on the Cp* coordinated systems. The magnetic moment for the more stable isomer is 1, varying to 3 in an interval of few eV. The coordination of the Cp* ligand contributes to the stabilization of the Ni atoms on the surface and the unpaired electrons stabilization, as see on the quadruplet isomers on the interval smaller than 1 eV from the lowest energy isomers.

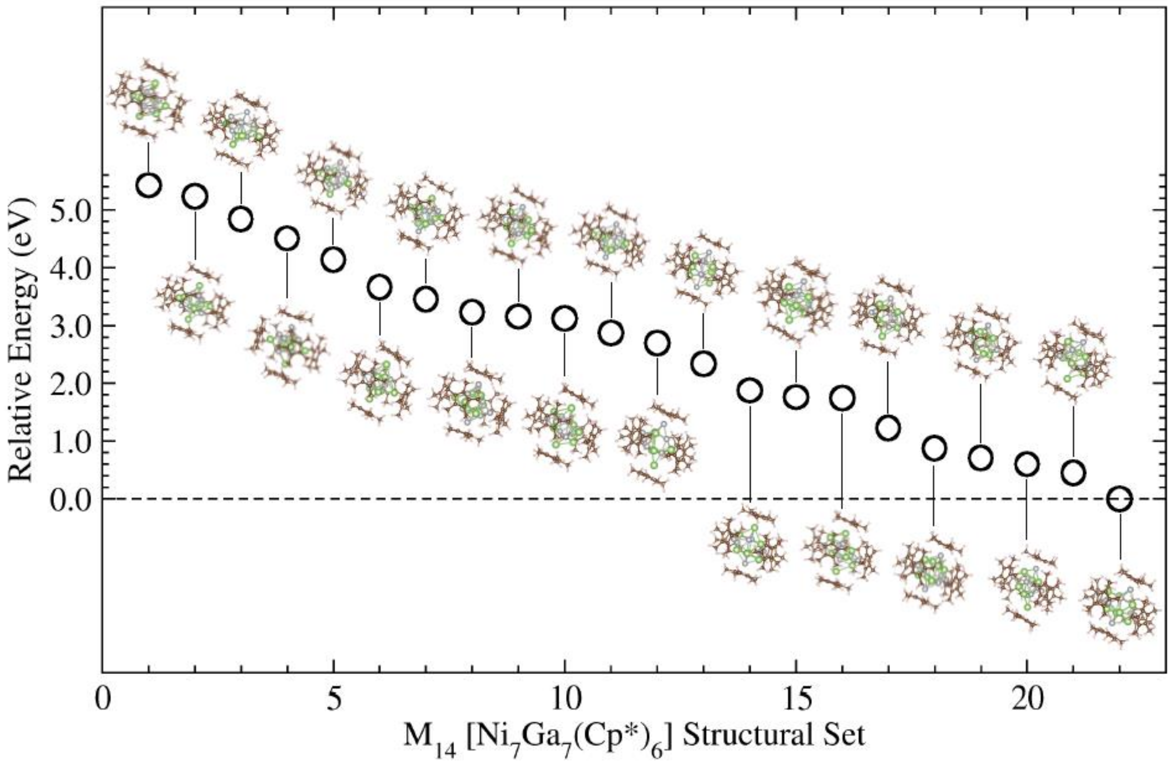

Figure S43. All calculated configurations for the $[Ni_7Ga_7(Cp^*)_6]$ systems.

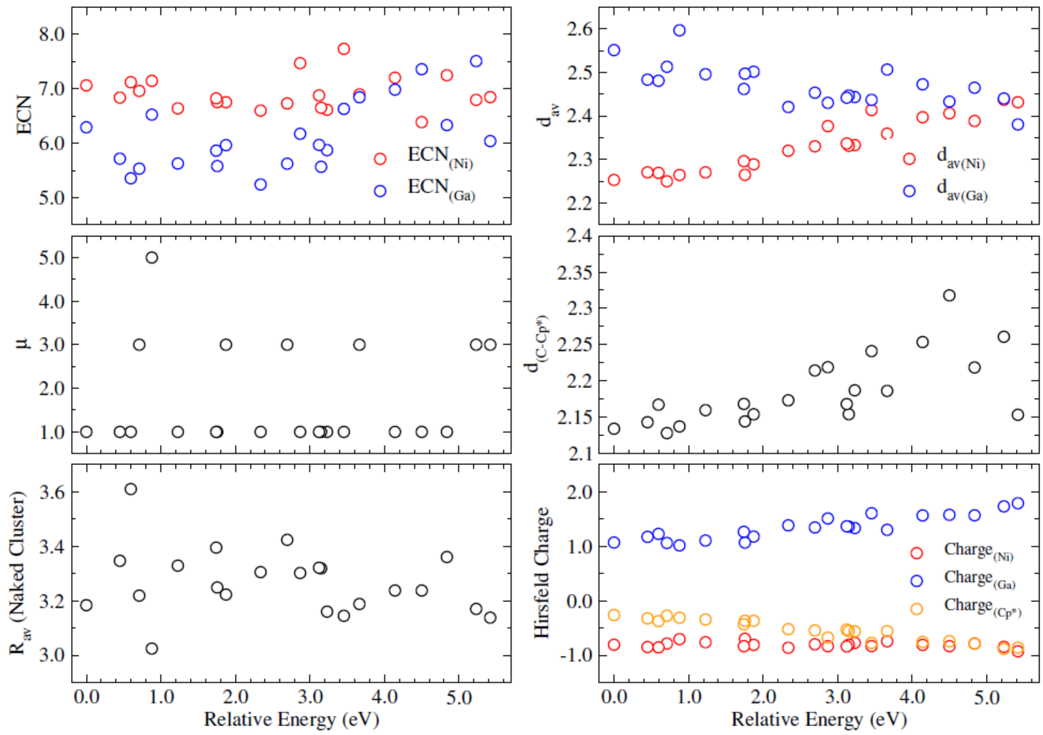

Figure S44. Geometrical and electronic analyses for the protected $[Ni_7Ga_7(Cp^*)_6]$ clusters (only metal atoms): average effective coordination number, ECN, in number of nearest neighbor (NNN), average weighted bond length, d_{av}, in Å, average cluster radius, R_{av}, in Å, and dipole moment, μ, in Debye, chemical ordering parameter, σ, and effective Hirsfeld charge, e.

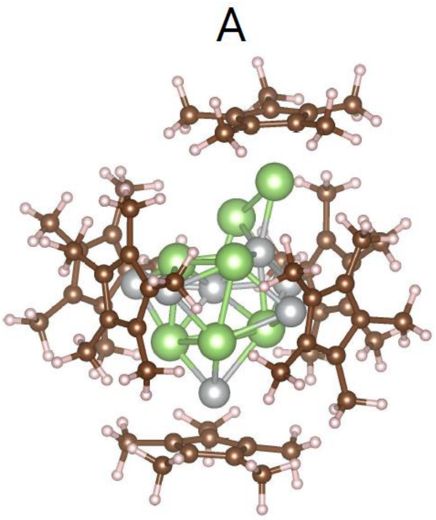

Figure S45. Lowest energy configurations obtained for the protected [$Ni_7Ga_7(Cp^*)_6$] systems obtained from the structures based on the unary systems (A) and on the experimental XRD structure (B).

Ni_7Ga_6 and Ni_6Ga_7

Figure S46 shows all configurations optimized by the FHI-aims package for the protected Ni_7Ga_6 clusters, i.e., 14 different configurations. From the results, the energy different between the highest and lowest energy configurations is about 4:0 eV, which can be explained by the location of the Ni atoms, i.e., those atoms bind to the ligands, as indicated in Figure S47. Furthermore, in Figure S48 of the most important geometric parameters for all calculated configurations, which can be seen clearly by the preference of the Ni atoms for high-coordination sites (supported by the effective coordination number). Similar results are also observed for the protected Ni_6Ga_7 clusters.

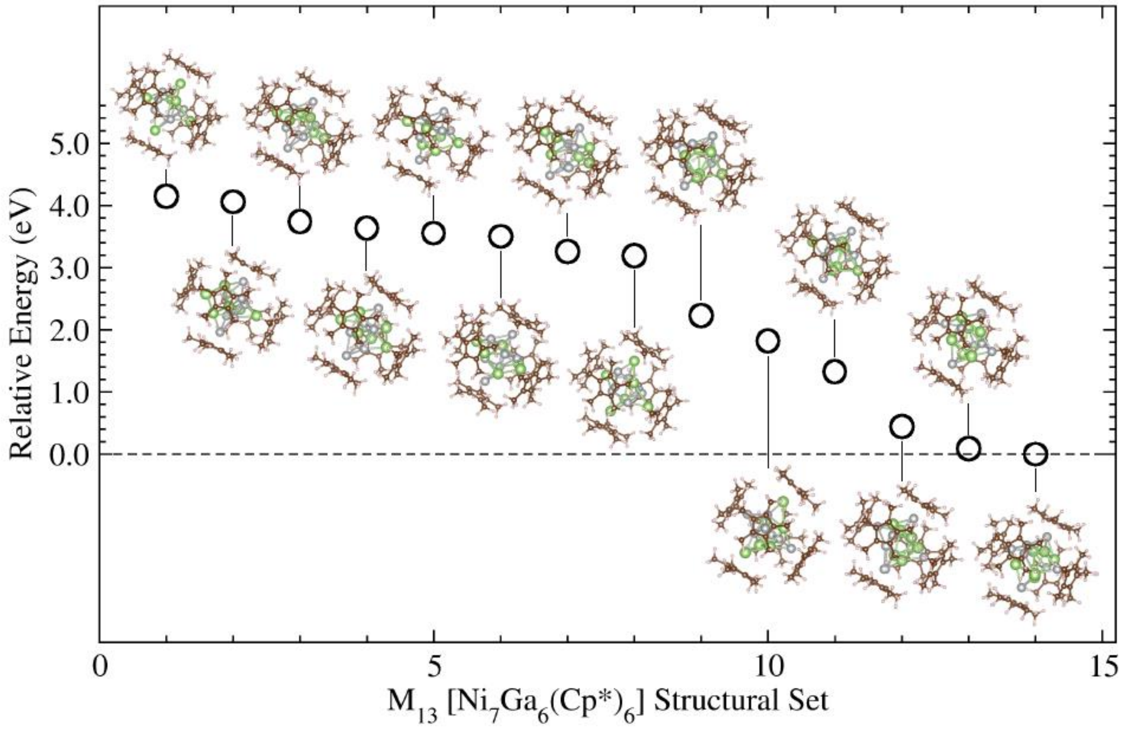

Figure S46. Structural configurations optimized for the protected [$Ni_7Ga_7(Cp^*)_6$] clusters.

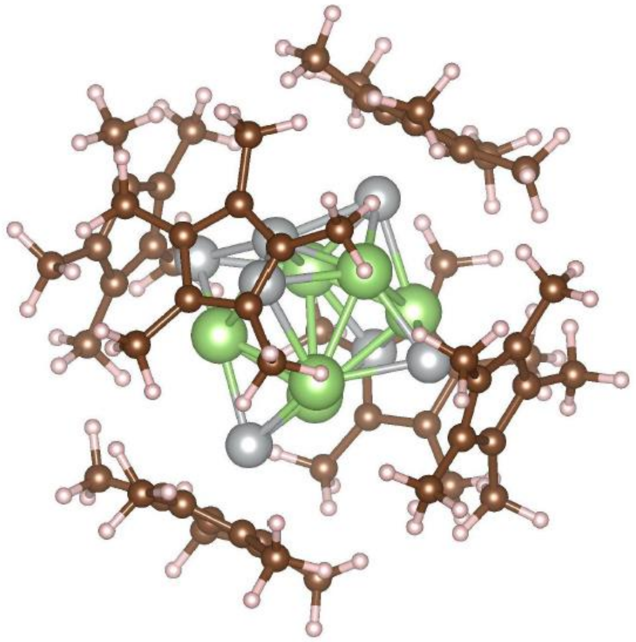

Figure S47. Lowest energy configuration for the protected [$Ni_7Ga_6(Cp^*)_6$] systems.

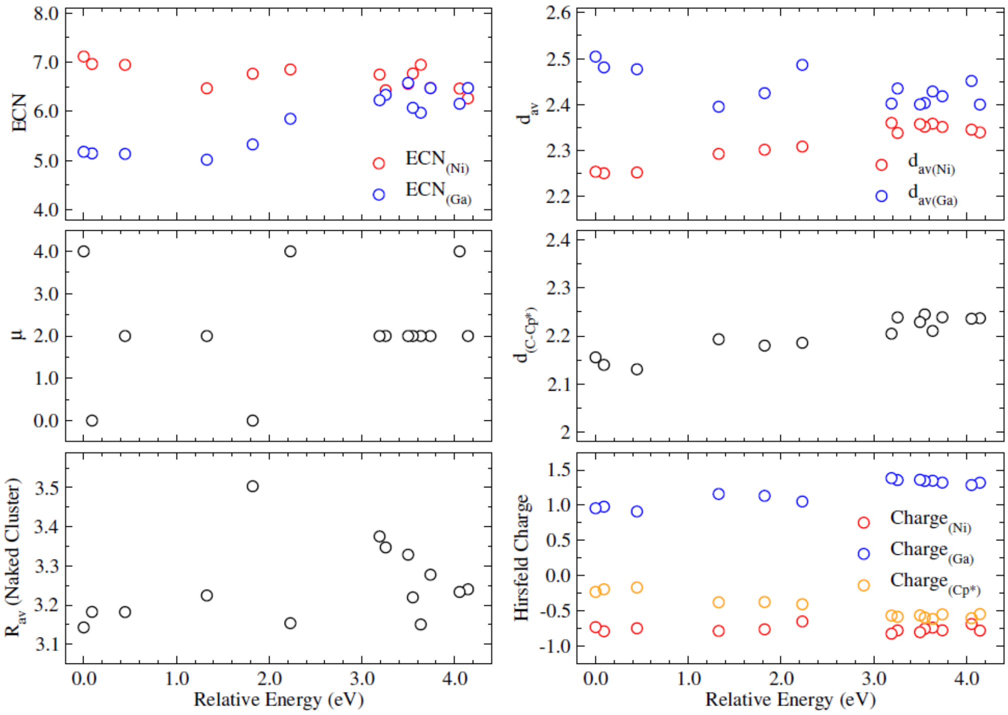

Figure S48. Geometrical and electronic analyses for the protected [$Ni_7Ga_6(Cp^*)_6$] clusters (only metal atoms): average effective coordination number, ECN, in number of nearest neighbor (NNN), average weighted bond length, d_{av}, in Å, average cluster radius, R_{av}, in Å, and dipole moment, μ, in Debye, chemical ordering parameter, σ, and effective Hirsfeld charge, *e*.

Ni_6Ga_7

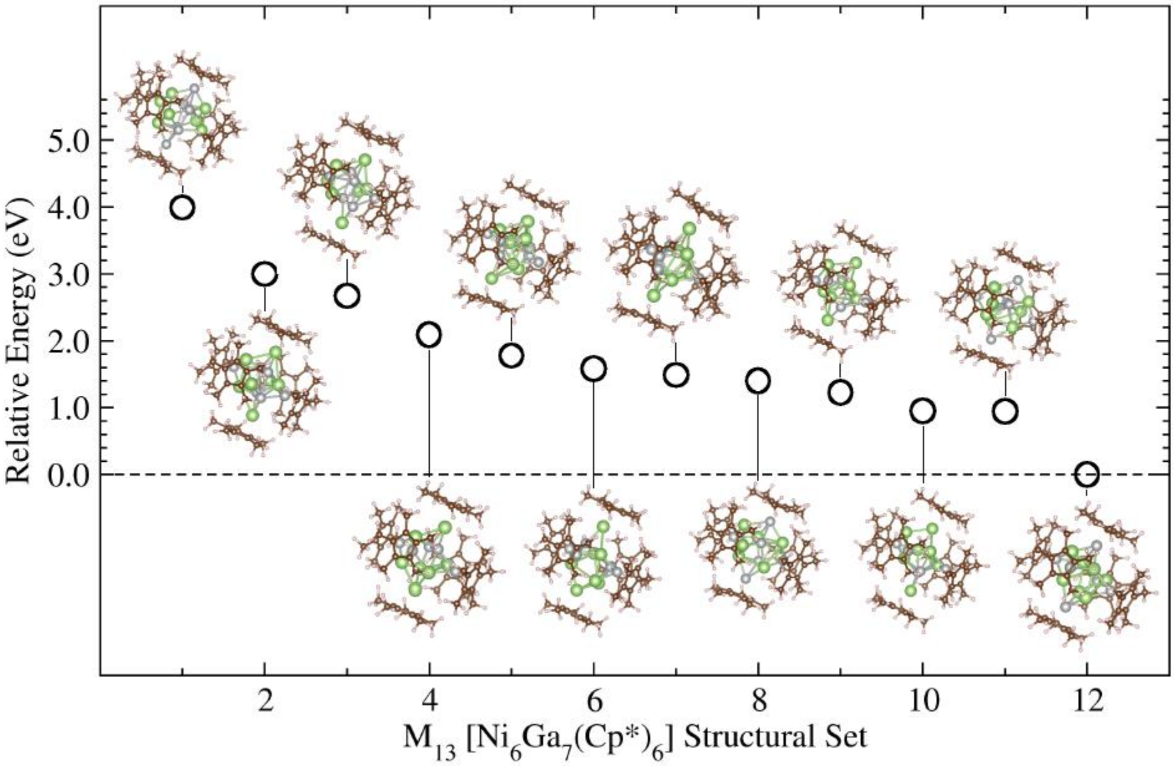

Figure S49. Structural configurations optimized for the protected [$Ni_6Ga_7(Cp^*)_6$] clusters.

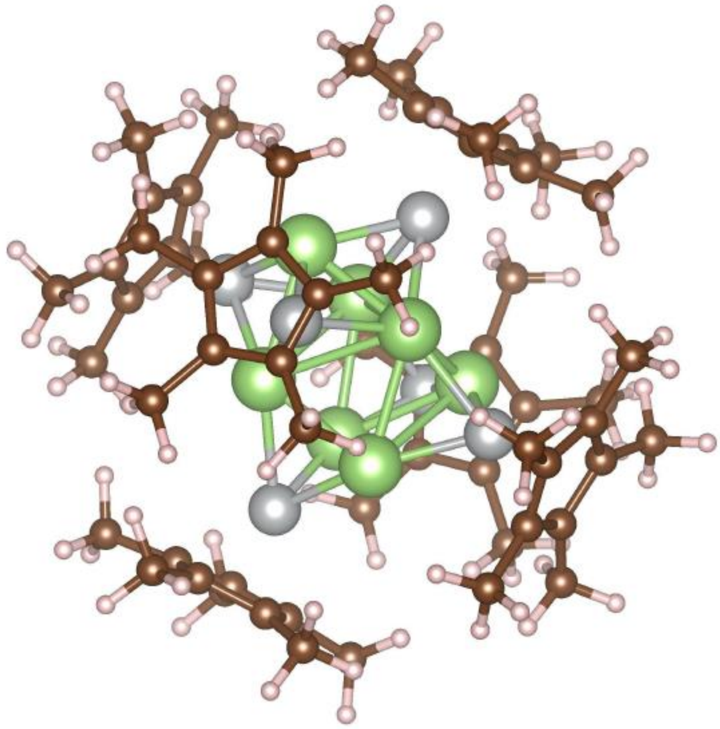

Figure S50. Lowest energy configuration for the protected [$Ni_6Ga_7(Cp^*)_6$] cluster.

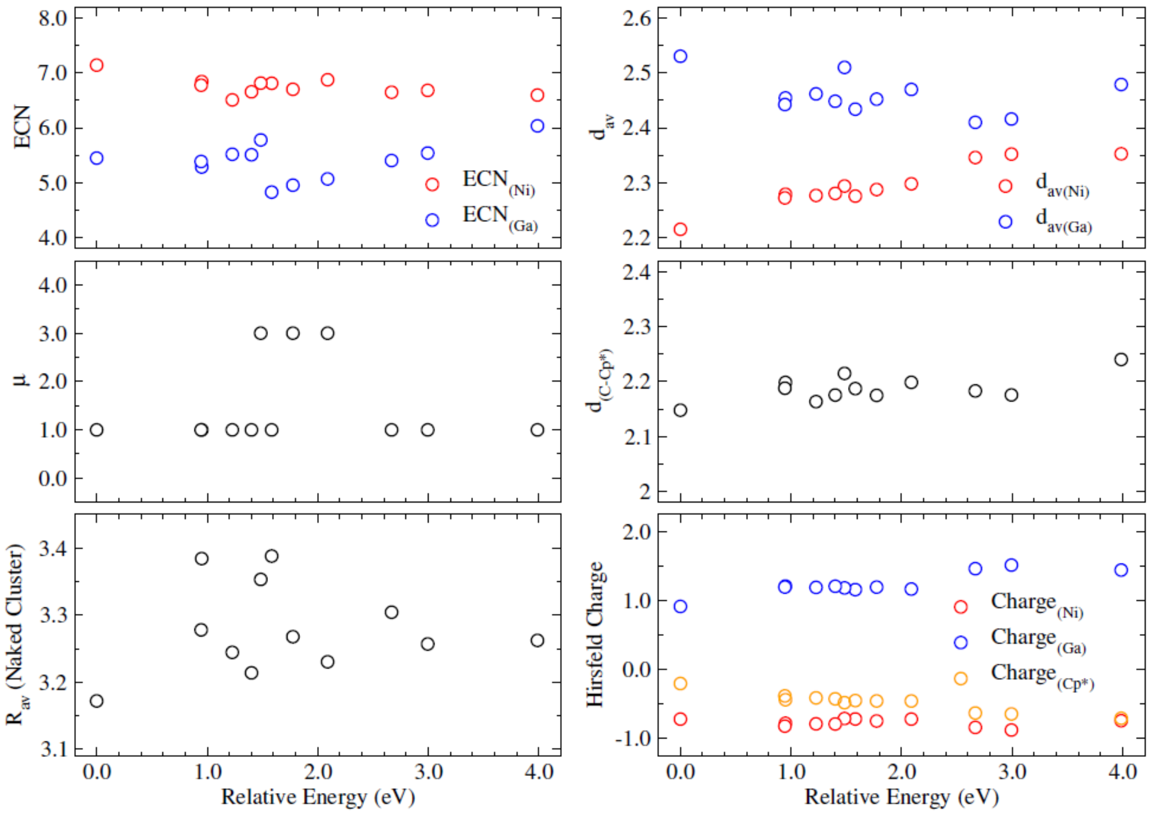

Figure S51. Geometrical and electronic analyses for the protected [$Ni_6Ga_7(Cp^*)_6$] clusters (only metal atoms): average effective coordination number, ECN, in number of nearest neighbor (NNN), average weighted bond length, d_{av}, in Å, average cluster radius, R_{av}, in Å, and dipole moment, μ, in Debye, chemical ordering parameter, σ, and effective Hirsfeld charge, e.

DFT calculations – Part 2

Bonding Details

Computational Details

Density Functional Theory (DFT) calculations[S8] were carried out with the use of the Amsterdam Density Functional code (ADF2017)[S9] with the addition of Grimme's D3 empirical corrections[S10] in order to consider dispersion effects. The triple-ξ Slater basis set plus two polarization functions (STO-TZP),[S11] was used, together with the Becke-Perdew (BP86)[S12-13] exchange-correlation functional. All the optimized structures were confirmed as true minima on their potential energy surface by analytical vibration frequency calculations. The NMR chemical shifts were computed according to the gauge-independent atomic orbitals (GIAO) method,[S14] assuming the Zero Order Regular Approximation (ZORA) for clusters with S = 1/2.[S15]

Table S25: HOMO-LUMO gaps ($\Delta_{H\text{-}L}$) and selected averaged interatomic distances (in Å) of the computed clusters. Ni_{exp} and Ga_{exp} designate "exposed" atoms.

	**$\Delta_{H\text{-}L}$ (eV)**	**Ni-Ga**	**Ga-Ga**	**Ni_{exp}-Ga**	**Ni_{exp}-Ni**	**Ga_{exp}-Ga**	**Ga_{exp}-Ni**
**$[Ga_6](NiC_p)_6$**	0.65	2.457	3.009	-	-	-	-
**$[NiGa_6](NiC_p)_6$**	0.69	2.452	2.907	2.349	2.771	-	-
**$\{[Ga_7](NiC_p)_6\}^+$**	0.72	2.451	2.923	-	-	3.131	3.298
**$[Ga_7](NiC_p)_6$**	-	2.471	2.873	-	-	3.124	3.289
**$[NiGa_7](NiC_p)_6$**	-	2.471	2.823	2.373	2.756	3.161	3.224
**$[Ni_2Ga_6](NiC_p)_6$**	0.71	2.471	2.844	2.359	2.762	-	-
**$[Ga_6](NiC_{p^*})_6$**	0.51	2.492	2.878	-	-	-	-
**$[NiGa_6](NiC_p{}^*)_6$**	0.55	2.482	2.798	2.412	2.761	-	_
**$\{[Ga_7](NiC_p{}^*)_6\}^+$**	0.55	2.500	2.851	-	-	3.117	3.079
**$[Ga_7](NiC_p{}^*)_6$**	-	2.523	2.817	-	-	3.123	3.076
**$[NiGa_7](NiC_p{}^*)_6$**	-	2.491	2.780	2.414	2.761	3.231	3.164
**$[Ni_2Ga_6](NiC_p{}^*)_6$**	0.55	2.499	2.802	2.395	2.756	-	-

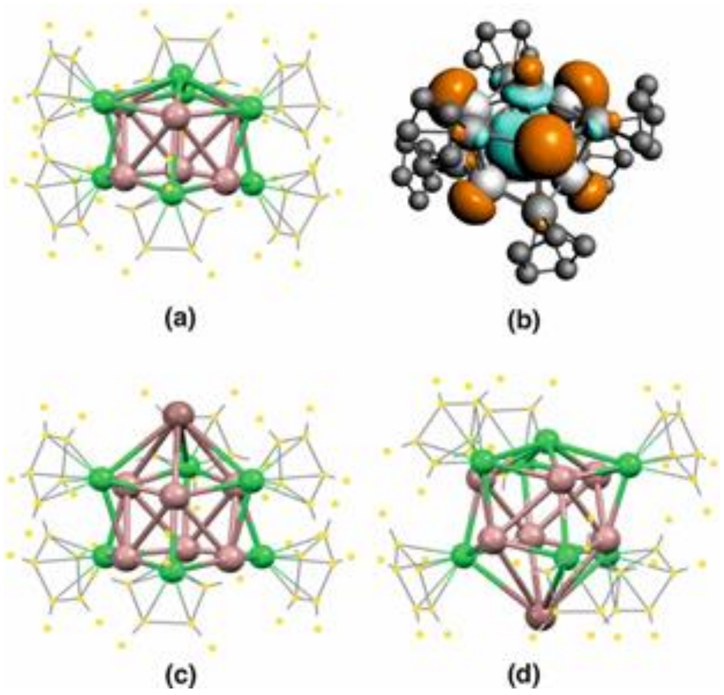

Figure S52. The optimized geometries of: (a) $[NiGa_6](NiCp)_6$, (b) LUMO of $[NiGa_6](NiCp)_6$ (c) $\{[Ga_7](NiCp)_6\}^+$ and (d) $[NiGa_7](NiCp)_6$.

Reactivity Tests: CO

While in [Ga_6]($NiCp^*$)$_6$ and [Ga_7]($NiCp^*$)$_6$ all nickel atoms are protected by a Cp* ligand, the M_{13} cluster [$NiGa_6$]($NiCp^*$)$_6$ and the M_{14} cluster [$NiGa_7$]($NiCp^*$)$_6$ exhibit an additional and exposed "active" nickel atom, without Cp* capping as part of the inner Ni/Ga core which is available for the Ni-CO coordination. For the M_{13} cluster [$NiGa_6$]($NiCp^*$)$_6$, however, the intermediate adduct complex [(CO)$NiGa_6$]($NiCp^*$)$_6$ is apparently not stable, leading to cluster degradation. We also assume that all clusters degrade over time in the presence of CO. Degradation products can be observed in ^1H, ^{13}C and ^{71}Ga NMR, IR spectroscopy and LIFDI-MS. The presence of free GaCp* (^1H NMR: 1.92ppm; ^{13}C NMR: 113 and 9.4 ppm; ^{71}Ga NMR: -650 ppm), $Ni(CO)_x$ (^{13}C: 192 ppm; IR) and one dominating pseudo-C_3 symmetric molecule probably assignable to $Ni(CO)_3$(GaCp*) or related species (^{13}C: 184 and 100 ppm, IR: 1950 and 1987 and 2066 cm^{-1}; ESI Figures S51-55). This is also reflected in the LIFDI-MS spectrum: several smaller species are observed and the main products of this reaction are the species [$(CO)_xNi_4Ga_4$]$(Cp^*)_4$ (x = 4, 5) and [$(CO)_yNi_4Ga_3$]$(Cp^*)_3$ (y = 1, 3, 5) (ESI, Figure S55-59).

NMR spectroscopy

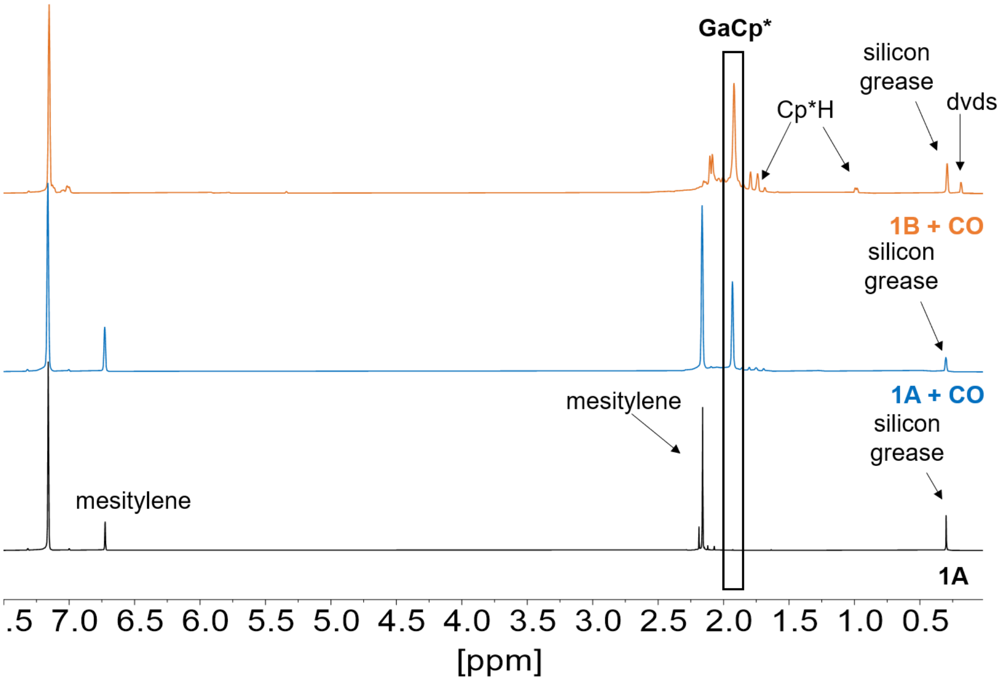

Figure S53. ^1H NMR spectra (toluene-d_8) of **1A** (major component **3**) and **1B** (major component **2**) upon exposure to CO showing a signal of free GaCp*. ^1H NMR (400 MHz, toluene-d$_8$, r.t.): δ_H *[ppm]* = 6.17 (s, mesitylene-C*H*), 2.17 (s, mesitylene-C*H*$_3$), 1.93 (GaCp*). The residues of dvds in **1B** originate from co-crystallized dvds.

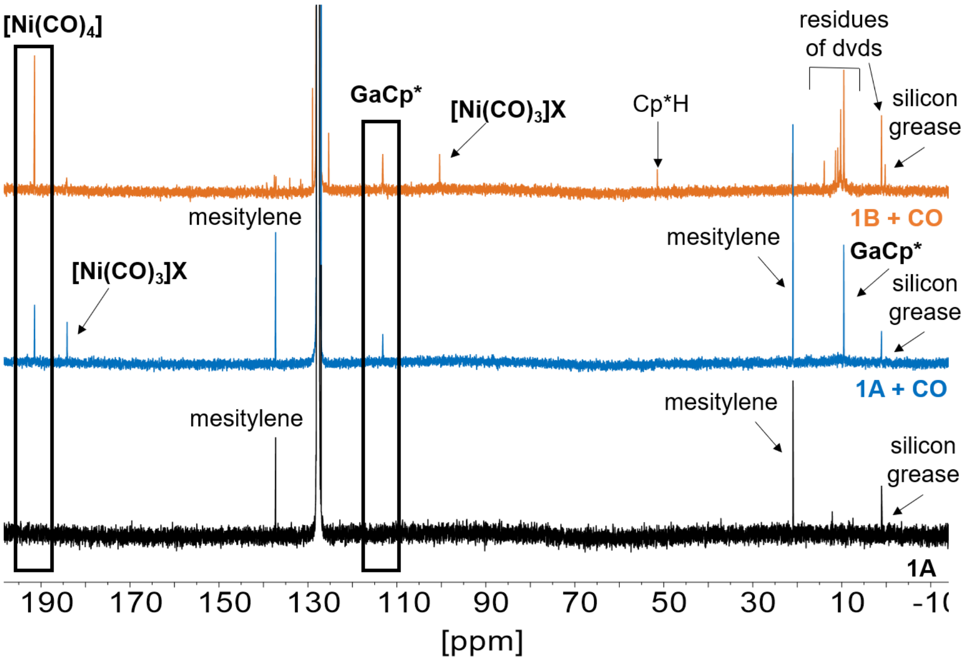

Figure S54. ^{13}C NMR spectra (toluene-d_8) of **1A** (major component **3**) and **1B** (major component **2**) upon exposure to CO showing a signal of $[Ni(CO)_4]$, free GaCp* and $[Ni(CO)_3]X$. ^{13}C NMR (400 MHz, toluene-d_8, r.t.): δ_C *[ppm]* = 192 (s, Ni(*C*O)$_4$), 137 (s, mesitylene-$C_{arom.}$), 113 (s, GaCp*, $C_{arom.}$), 20.9 (s, mesitylene-CH_3), 9.44 (s, GaCp*, CH_3). The residues of dvds in **1B** originate from co-crystallized dvds.

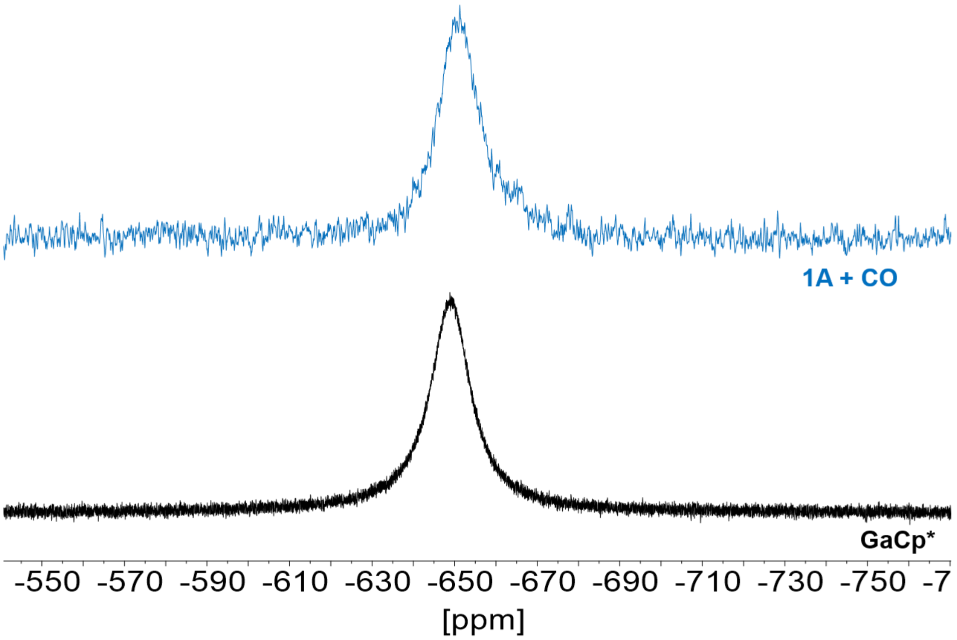

Figure S55. ^{71}Ga NMR spectra (toluene-d_8) of **1A** (major component **3**) and **1B** (major component **2**) upon exposure to CO (blue, top) showing a signal of free GaCp* (see reference, black, bottom). ^{71}Ga NMR (400 MHz, toluene-d_8, r.t.): δ_{Ga} *[ppm]* = -650 (*Ga*Cp*).

IR spectroscopy

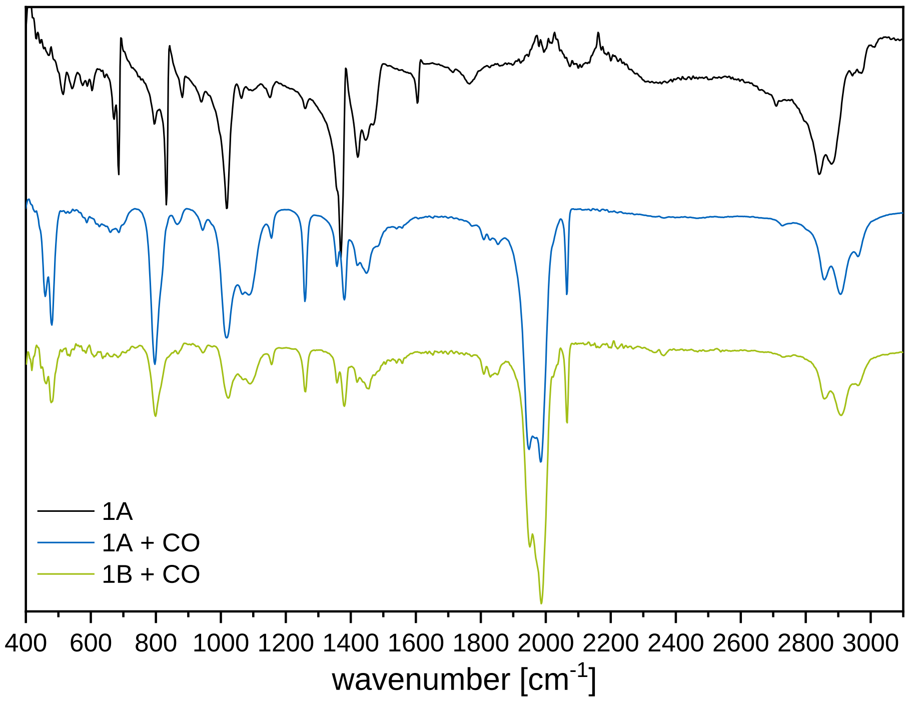

Figure S56. FT-IR measurements of **1A** (major component **3**) and **1B** (major component **2**) with CO. IR (ATR, neat, cm^{-1}): 2960 (w), 2900 (m), 2857 (m), 2066 (s), 1987 (s), 1950 (s), 1851 (w), 1829 (w), 1808 (w), 1483 (w), 1448 (w), 1380 (w), 1357 (m), 1258 (s), 1155 (w), 1089 (m), 1067 (m), 1021 (s), 945 (s), 863 (w), 799 (w), 685 (w), 659 (w), 626 (w), 587 (w), 480 (m), 459 (s).

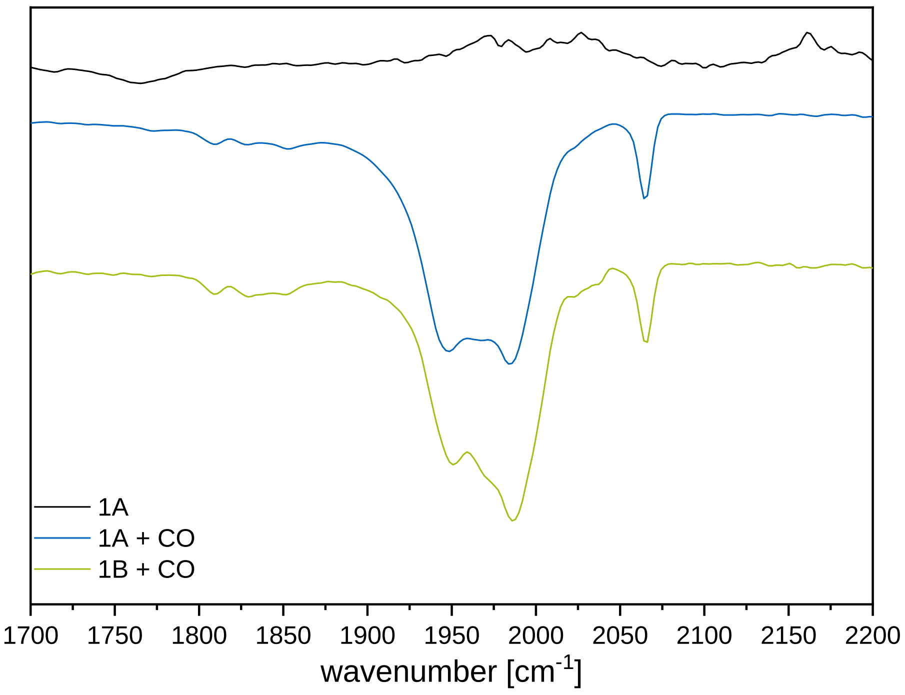

Figure S57. Representative cutout of FT-IR measurements of **1A** (major component **3**) and **1B** (major component **2**) upon exposure to CO showing new signals which can be assigned to species with a X-Ni$(CO)_3$ structure.

LIFDI-MS measurements

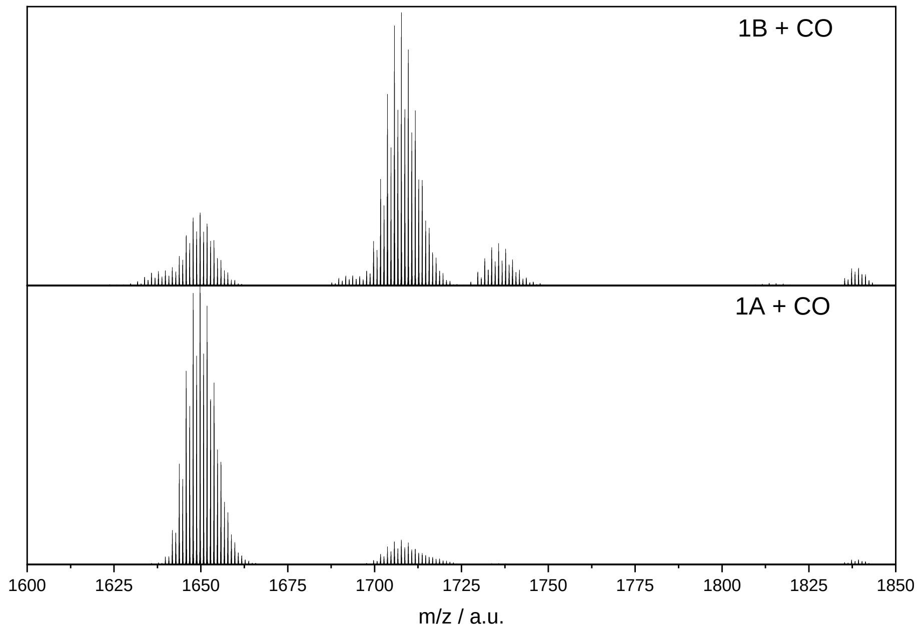

Figure S58. Representative cutout of LIFDI-MS measurements of **1A** (major component **3**, bottom) and **1B** (major component **2**, top) upon exposure to CO showing the new signals $[Ni_7Ga_7](Cp^*)_6CO$ (m/z = 1736.9) and $[Ni_8Ga_8](Cp^*)_6$ (m/z = 1837.3). MS (LIFDI-TOF, toluene): m/z = 1165.9 ($[Ni_6Ga_2Cp^*_5]^+$, calc. 1167.1), 1193.8 ($[Ni_6Ga_2Cp^*_5CO]^+$, calc. 1195.1), 1637.8 ($[Ni_7Ga_6Cp^*_6]^+$, calc. 1638.6), 1649.8 ($[Ni_6Ga_7Cp^*_6]^+$, calc. 1650.3), 1706.7 ($[Ni_7Ga_7Cp^*_6]^+$, calc. 1706.2), 1734.4 ($[Ni_6Ga_7Cp^*_6CO]^+$, calc. 1734.2), 1835.9 ($[Ni_6Ga_9Cp^*_6]^+$, calc. 1836.7).

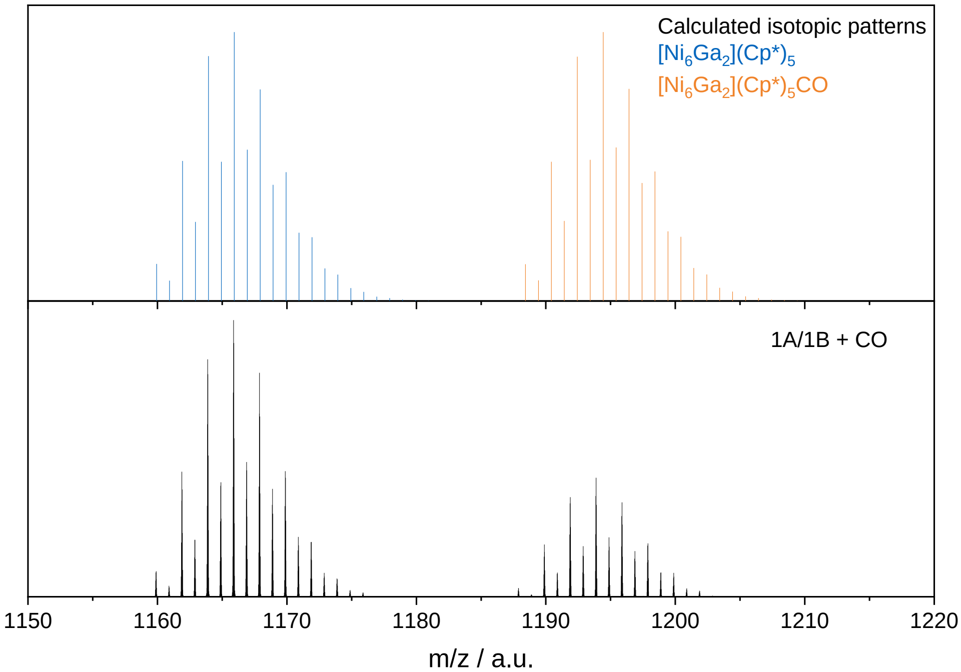

Figure S59. Representative cutout and zoom-in of LIFDI-MS spectra of **1** upon exposure to CO showing the new signals $[Ni_6Ga_2]($ $Cp^*)_5$ (m/z = 1167.1) and the corresponding CO adduct $[Ni_6Ga_2](Cp^*)_5CO$ (m/z = 1195.1). Both **1A** (major component **3**) and **1B** (major component **2**) show identical signals in this region.

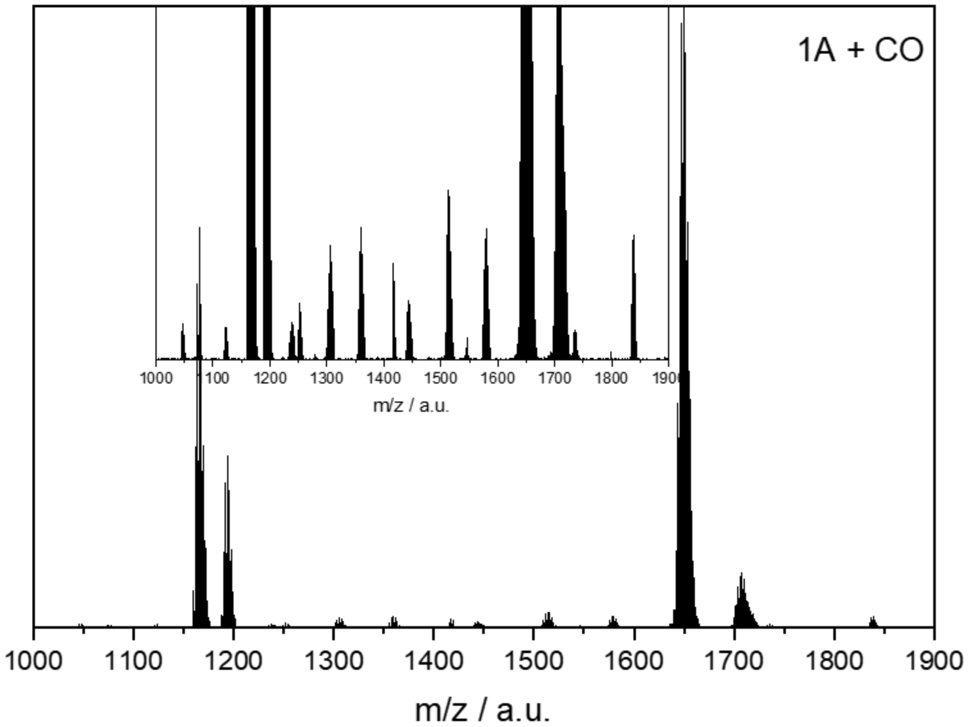

Figure S60. Full LIFDI mass spectra of cluster mixture **1A** (major component **3**) after treatment with CO zoom-in for a better identification of small cluster signals.

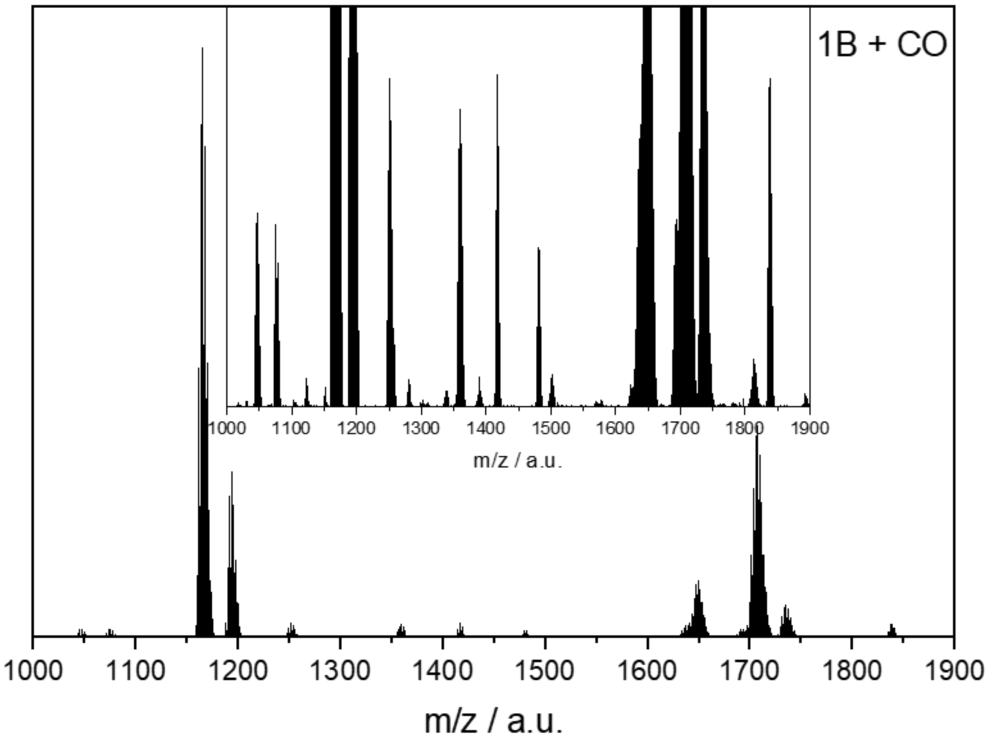

Figure S61. Full LIFDI mass spectra of cluster mixture **1B** (major component **2**) after treatment with CO with zoom-in for a better identification of small cluster signals.

Supplementary References

[S1] B. Bogdanović, M. Kröner, G. Wilke, *Liebigs Ann. Chem.* **1966**, 699, *1*, 1-23.

[S2] P. Jutzi, L. O. Schebaum, *J. Organomet. Chem.* **2022**, 654, *1*, 176-179.

[S3] M. Muhr, J. Hornung, J. Weßing, C. Jandl, C. Gemel, R. A. Fischer, *Inorg. Chem.* **2020**, 59, *7*, 5086-5092.

[S4] Bruker AXS Inc., *APEX suite of crystallographic software, APEX 3 Version 2015-5.2*, Madison, Wisconsin, USA, **2015.**

[S5] Bruker AXS Inc., *SAINT, Version 8.34A and SADABS, Version 2014/5*, Madison, Wisconsin, USA, **2014.**

[S6] G. Sheldrick, *Acta Crystallogr., Sect. A* **2008,** *64*, 112-122.

[S7] C. B. Hubschle, G. M. Sheldrick, B. Dittrich, *J. Appl. Crystallogr.* **2011,** *44*, 1281-1284.

[S8] a) G. Sheldrick, *Acta Crystallogr., Sect. A* **2015,** *71*, 3-8. b) G. Sheldrick, *Acta Crystallogr., Sect. C* **2015,** *71*, 3-8.

[S9] *International Tables for Crystallography, Vol. C* (Ed.: A. J. Wilson), Kluwer Academic Publishers, Dordrecht, The Netherlands, **1992**, Tables 6.1.1.4 (pp. 500–502), 4.2.6.8 (pp. 219–222), and 4.2.4.2 (pp. 193–199).

[S10] C. F. Macrae, I. J. Bruno, J. A. Chisholm, P. R. Edgington, P. McCabe, E. Pidcock, L. Rodriguez-Monge, R. Taylor, J. van de Streek, P. A. Wood, *J. Appl. Cryst.* **2008**, *41*, 466–470.

[S11] Parr, R. G.; Yang, W. *Density-Functional Theory of Atoms and Molecules*. **1994**, Oxford University Press, UK.

[S12] a) G. te Velde, F. M. Bickelhaupt, S. J. A. van Gisbergen, C. F. Guerra, E. J. Baerends, J. G. Snijders, T. Ziegler, *J. Comput. Chem.* **2001**, *22*, 931-967; b) ADF2016, SCM, *Theoretical Chemistry*, Vrije Universiteit: Amsterdam, The Netherlands; http://www.scm.com.

[S13] S. Grimme, *J. Comput. Chem.* **2006**, *27*, 1787–1799.

[S14] E. V. Lenthe, E. J. Baerends, *J. Comput. Chem.* **2003**, *24*, 1142–1156.

[S15] A. D. Becke, *Phys. Rev. A* **1988**, *38*, 3098–3100.

[S16] J. P. Perdew, *Phys. Rev. B* **1986**, *33*, 8822–8824.

[S17] G. Schreckenbach, T. Ziegler, *J. Phys. Chem.* **1995**, *99*, 606-611.

[S18] G. J. Snijders, E. J. A. Baerends, *Mol. Phys.* **1978**, *36*, 1789-1804.

Author Contributions

The manuscript was written through contributions of all authors. All authors have given approval to the final version of the manuscript.
